# A replenishable peritoneal implant for localized delivery and peritoneal fluid sampling in ovarian cancer

**DOI:** 10.1016/j.device.2026.101050

**Published:** 2026-03-20

**Authors:** Aoibhin M. Sheedy, Mihir Shetty, Anna Weis, Laura E. Bendzick, Terran Stenger, Zhenya Ni, Philippa R. Kennedy, Jacob A. Myers, Niamh Ward, Lesley Trask, Hannah Prendeville, Joanne O’Dwyer, Michael O’Dwyer, Ellen T. Roche, Garry P. Duffy, Jeffrey S. Miller, Melissa A. Geller, Eimear B. Dolan, Martin Felices

**Affiliations:** 1 Biomedical Engineering, School of Engineering, College of Science and Engineering, University of Galway, Galway, Ireland; 2 CÚRAM, Centre for Research in Medical Devices, University of Galway, Galway, Ireland; 3 Masonic Cancer Center, University of Minnesota, Minneapolis, MN 55455, USA; 4 Department of Obstetrics, Gynecology and Women’s Health, University of Minnesota, Minneapolis, MN, USA; 5 Department of Medicine, University of Minnesota, Minneapolis, MN 55455, USA; 6 Anatomy and Regenerative Medicine Institute (REMEDI), School of Medicine, University of Galway, Galway, Ireland; 7 Pharmacy, School of Pharmacy and Medical Sciences, University of Galway, Galway, Ireland; 8 Apoptosis Research Centre, University of Galway, Galway, Ireland; 9 Institute for Medical Engineering and Science, Massachusetts Institute of Technology, Cambridge, MA 02142, USA; 10 Department of Mechanical Engineering, Massachusetts Institute of Technology, Cambridge, MA 02139, USA; 11 Wyss Institute of Biologically Inspired Engineering, Boston, MA 02215, USA; 12 Senior author; 13 These authors contributed equally; 14 Lead contact

## Abstract

Intraperitoneal (i.p.) therapy improves outcomes in abdominal cancers but remains underutilized due to complications from repurposed catheters. In this work, we present a replenishable therapeutic implant for repeated, localized delivery of therapies to the peritoneal cavity. In an ovarian cancer mouse model, expanded natural killer (eNK) cells were delivered once weekly and interleukin-15 (IL-15) thrice weekly. This regime reduced tumor burden compared to standard i.p. injection. The implant supports co-administration of chemotherapy, cytokines, monoclonal antibodies, or other protein-based therapies. Negative pressure applied via the port enabled longitudinal sampling of peritoneal fluid without additional surgical intervention. By reducing procedural burden and improving adaptability, the implant can help increase patient retention and therapeutic efficacy in ovarian cancer and other intra-abdominal cancers.

## In brief

A.M. Sheedy et al. present a replenishable implant that enables repeated delivery of therapies directly to the peritoneum for ovarian cancer treatment. This minimally invasive platform improves therapeutic efficacy and allows longitudinal fluid sampling to monitor disease response.

## Graphical abstract

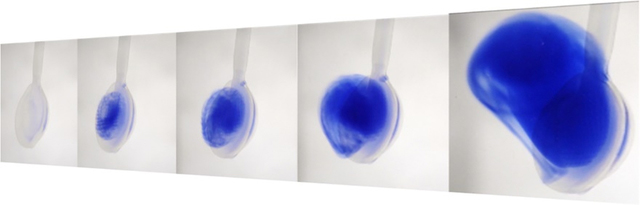

## INTRODUCTION

Localized delivery of therapeutic agents such as small-molecule drugs, biologics, and cell therapies enhances therapeutic efficacy while minimizing side effects compared with systemic delivery.^1–3^ This is critical for cytotoxic therapeutic agents, such as chemotherapies, where off-target effects can cause complications, including peripheral neuropathy and extensive renal damage.^4^ For non-metastatic cancers, localized treatment could improve outcomes while minimizing adverse side effects associated with intravenous (i.v.) chemotherapy.^5^ Repeated localized intraperitoneal (i.p.) delivery of platinum-based chemotherapies has improved outcomes in ovarian cancer.^6–8^ Ovarian cancer, a malignancy originating in the ovaries or fallopian tubes,^9^ is commonly detected at advanced stages because of nonspecific symptoms such as back pain, bloating, or constipation.^10^ Five-year survival rates decrease to 46% and 26% for stage III and stage IV disease, respectively.^11^ However, even at these advanced stages, the disease predominantly resides within the i.p. space,^12^ making localized therapeutic intervention the optimal choice.

Advances in i.p. chemotherapy for ovarian cancer were demonstrated in three pivotal clinical trials over 20 years,^6–8^ which compared i.p. with i.v. chemotherapy in stage III patients.^6–8^ The GOG172^8^ trial reported a 16-month increase in median overall survival when i.p. delivery was compared with i.v. chemotherapy,^6–8^ leading to the National Cancer Institute recommending i.p. chemotherapy as the preferred delivery route for advanced-stage ovarian cancer.^13^ A meta-analysis indicated a 21.6% decrease in risk of death with i.p. chemotherapy compared with i.v. (hazard ratio: 0.78; 95% confidence interval: 0.69–0.89).^14^ The success of i.p. chemotherapy is likely attributed to higher drug concentrations and a longer drug half-life within the peritoneal cavity.^15^

Despite the promising data, fewer than 50% of eligible patients receive i.p. chemotherapy.^16^ At present, there are no devices on the market built solely with the intent to deliver long-term i.p. therapy, highlighting an untapped opportunity for improved patient outcomes. Repurposed catheters were used in the three pivotal trials^6–8^ mentioned earlier. For many patients, the frequency and severity of catheter-related issues lead to treatment discontinuation; for example, Walker et al. found that only 42% of patients (50/119) completed the prescribed six courses of i.p. treatment. Catheter complications (catheter blockages, leaking, infections, and port access problems) accounted for 34% of patients (40/119) discontinuing i.p. treatment.^17^ A recent randomized trial with equivalent dosing between i.p. and i.v. chemotherapy arms has shown comparable outcomes,^18^ suggesting limited additional benefit for i.p. delivery in the chemotherapy setting. However, i.p. access is likely to become increasingly critical for the safe and effective administration of emerging immunotherapies and gene-based treatments. This is highlighted in a phase 1 study of i.p. nivolumab after cytoreductive surgery and hyperthermic intraperitoneal chemotherapy (HIPEC),^19^ where 4/17 patients (23.5%) discontinued early due to peritoneal catheter complications, despite good drug tolerability, highlighting that the delivery system remains a major barrier. A translational analysis iPocc phase 3 trial^19^ demonstrated that patients with “immune hot” tumors (higher T cell/natural killer [NK] cell/cytotoxicity signatures) had better outcomes with i.p. vs. i.v. delivery. Further support for the therapeutic power of i.p. delivery has been demonstrated in a clinical trial, OVATION-2,^20^ using IMNN-001 (Imunon), an interleukin (IL)-12 DNA plasmid vector administered i.p. with neoadjuvant/adjuvant chemotherapy. In a phase 1/2 trial, IMNN-001 was associated with a 13-month survival benefit, prompting advancement into a phase 3 study. These observations highlight the potential of i.p.-delivered cytokine-based approaches to fundamentally alter the therapeutic landscape of ovarian cancer. Repurposed catheters, such as the Tenckhoff i.p. catheter (Medtronic), the Celsite Implantofix Access Port System (B Braun), and the PowerPort Port-a-Cath (Bard), summarized in Table S1, have been used despite reported complications.^17,21–24^

Cell immunotherapies, such as chimeric antigen receptor T cell (CAR T), have revolutionized the treatment options for hematological cancers^25^ but remain less effective against solid tumors, such as ovarian cancer.^26^ NK cells are considered part of the innate immune system, and NK cell therapies have shown promise in ovarian cancer treatment.^1,27–29^ Studies have reported a positive correlation between NK cell proportions within ascites and overall survival.^30^ Ascites is a buildup of fluid in the i.p. space that only occurs in disease. Geller et al.^27^ demonstrated that i.p.-administered NK cells, maintained with IL-2 or IL-15, reduced tumor burden in an ovarian cancer xenograft murine model and retained cytotoxicity over time. Similar positive results were reported elsewhere,^28,31,32^ but the same effect was not observed with i.v. delivery.^27^ Recent clinical trials investigating NK cell therapies against ovarian cancer (ClinicalTrials.gov: NCT02118285, NCT03213964, and NCT06342986) are using the i.p. delivery route and are also relying on repurposed catheters (brand not disclosed) despite the challenges outlined in Table S1. Cell immunotherapies are a living, expensive, and delicate therapeutic cargo, requiring precise delivery to tumors to avoid wastage and maximize therapeutic impact. Delivery methods must allow easy insertion, be safe, and enable targeted, repeated delivery to the tumor site. Additionally, peritoneal fluid can provide insights into disease progression,^33,34^ but it is not possible to routinely monitor this environment using existing treatment modalities. Collecting samples of cells from the peritoneal fluid during treatment could provide information for real-time therapy adjustments.

To address this clinical need, we designed a replenishable therapeutic peritoneal implant. Constructed from a flexible thermoplastic polymer, this implant facilitates i.p. therapy delivery in a murine model of ovarian cancer and permits minimally invasive peritoneal fluid collection to monitor the peritoneum, including the tumor microenvironment. This is the first time an implant, designed specifically for ovarian cancer, has been developed to achieve both effective repeated local therapy delivery and sampling in this capacity. The design supports co-administration of cellular therapies and other therapeutics, including, but not limited to, chemotherapy, cytokines, monoclonal antibodies, or other therapeutic proteins. The design represents a versatile and tunable mono-material platform with clinical potential for other diseases that require long-term, repeated delivery of therapeutics to the peritoneal cavity.

## RESULTS

### Design and *in vitro* assessment of the peritoneal implant

**Figure 1.**
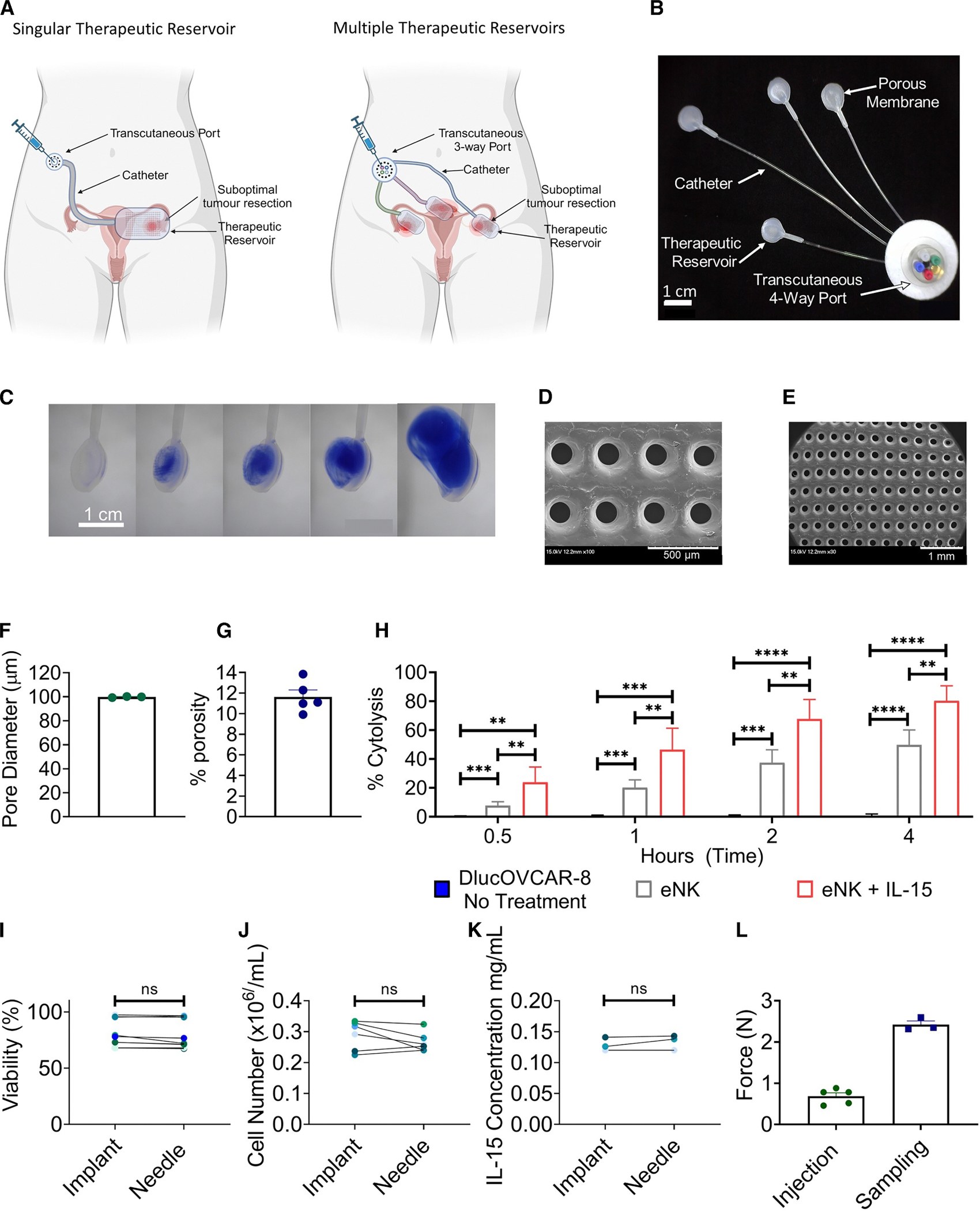
Replenishable therapeutic implant conceptualization, manufacture, and *in vitro* testing (A) Schematic of two versions of the proposed design. A single therapeutic reservoir or multiple therapeutic reservoirs placed over areas of residual tumor in the intraperitoneal space. (B) A prototype of the therapeutic implant with four porous reservoirs connected to a single transcutaneous port. Scale bar: 1 cm. (C) Delivery of a model drug, trypan blue, from the therapeutic implant. Scale bar: 1 cm. (D and E) Representative scanning electron microscopy (SEM) images (×180 magnification at 15.0 kV) of the porous membrane showing 100-μm pores. Scale bars: 500 μm (D) and 1 mm (E). (F and G) Quantification of (F) pore diameter (*n* = 3 porous membranes) and (G) percentage porosity (*n* = 5 membranes). (H) Analysis of 2:1 eNK and eNK + IL-15 (effector) to target (OVCAR-8) cell killing (percentage of cytolysis of OVCAR-8 cells) at 0.5, 1, 2, and 4 h using the xCELLigence RTCA system. *n* = 4 donors. (I and J) Cell (I) viability or (J) number of eNK cells after delivery through implant or 32G needle. *n* = 3 donors and *n* = 3 implants. (K) Concentration of IL-15 delivered through the implant or 32G needle. *n* = 3 implants. (L) Forces required to deliver through (*n* = 5 implants) and sample from (*n* = 3 implants) the implant under normal conditions. eNK, expanded natural killer cells; D-luc+OVCAR-8, human ovarian cancer cell line OVCAR-8 transfected with D-luciferase; IL-15, interleukin-15. Data are presented as mean ± standard error of mean. ns = *p* > 0.05, **p < 0.01, ***p < 0.001, ****p < 0.0001.

The implant is fabricated from a compliant, flexible material that conforms to the peritoneal environment, incorporates defined porosity to enable diffuse therapy release, and supports more homogeneous distribution throughout the cavity. It was designed to overcome limitations of catheter-based systems that suffer from mechanical mismatch in the surrounding tissue, limited porosity, non-uniform therapeutic distribution within the peritoneal cavity, and complications (such as leakage, blockage, displacement, and perforation). The implant incorporates a transcutaneous port, connected to one or multiple reservoirs via indwelling catheters (Figures 1A and 1B). The reservoirs include a semipermeable membrane (Figures 1B–1E), which allows the therapeutic cargo to be dispensed into the target tissue. Multiple reservoirs can target disease at several distinct sites within the peritoneal cavity simultaneously, enabling therapeutic and sampling impact from just one transcutaneous port. The transcutaneous port and the indwelling catheter facilitate multiple minimally invasive replenishments of a range of therapeutics directly to the target site. Similarly, applying a negative pressure to the transcutaneous port can allow for repeated and minimally invasive collection of peritoneal samples to enable real-time monitoring of the therapeutic response. In this study, we investigated the feasibility of a single therapeutic reservoir with a catheter and transcutaneous port to deliver to and collect cells from the peritoneal space of NOD.Cg-Prkdc^scid^ Il2rg^tm1Wjl^/SzJ (NSG) mice. The mono-material implant was manufactured using thermoplastic polyurethane (TPU), selected to minimize immunogenicity and drug absorption or retention. Briefly, laser-cut porous TPU membranes (11.62% ± 1.34% porosity, average pore diameter: 99.81 ± 0.37 μm, Figures 1D–1G) were heat sealed to a thermoformed hemispherical TPU reservoir. A Micro-Renathane TPU catheter connected to a transcutaneous port (VABM1B/22, Instech Laboratories) was inserted between these components to form a single assembly.

To model therapeutic delivery of cells and cytokines in the setting of ovarian cancer, we first tested the cytolytic potential of NK cell products (expanded [e]NK cells), treated with or without IL-15, against ovarian cancer cells using an impedance-based cytolytic platform. Unlike T cells, NK cells can be safely used in allogeneic settings.^35^ eNK cells are NK cells that have been grown and activated, with engineered feeder cells *ex vivo*, to increase their numbers in order to produce therapeutically relevant doses before being reintroduced into a patient for therapeutic purposes.^36^ IL-15 is critical for NK cell survival, proliferation, and cytotoxic priming to respond to abnormal cells.^37^ The cytotoxicity of eNK cells ± IL-15 was evaluated *in vitro* using the human ovarian cancer cell line OVCAR-8 transfected with D-luciferase (D-luc+OVCAR-8, NCI). eNK cells at a ratio of 2:1 eNK:D-luc+OVCAR-8 significantly increased D-luc+OVCAR-8 cytolysis relative to untreated controls after 0.5 (*p* < 0.001), 1 (*p* < 0.001), 2 (*p* < 0.001), and 4 (*p* < 0.0001) h of treatment, indicating that eNK cells can effectively induce D-luc+OVCAR-8 cytolysis. The cytolytic capacity of eNK cells was improved with the addition of 10 nM IL-15, with significant differences observed after 0.5 (*p* < 0.01), 1 (*p* < 0.01), 2 (*p* < 0.01), and 4 (*p* < 0.01) h of treatment in comparison with treatment with eNK cells alone (Figure 1H). Hence, eNK cells + IL-15 were selected as the therapy to be delivered through our therapeutic peritoneal implant *in vivo*.

Next, we compared the efficacy of delivering eNK cells + IL-15 through our implant *in vitro*. A syringe containing the therapeutic cargo was connected to the distal transcutaneous port via a 25G PinPort injector (PNP3M, Instech). We compared eNK cell + IL-15 delivery through our implant to delivery by i.p. injection (32G needle), the current gold standard for i.p. therapy delivery in murine models.^27,28,38,39^ eNK cell viability and delivered cell number were not significantly different when delivered through the implant compared with a 32G needle. An average viability of 80.85% ± 11.38% was observed when cells were delivered through the implant compared with 79.41% ± 12.07% when injected through a 32G needle (Figure 1I). An average of 0.289 ± 0.043 × 10^6^ cells/mL was delivered through the implant compared with 0.266 ± 0.029 × 10^6^ cells/mL through the needle (Figure 1J). Similarly, the concentration of IL-15 delivered via the implant was not significantly different from that following delivery through a 32G needle (0.134 ± 0.010 vs. 0.129 ± 0.009 mg/mL, Figure 1K). Additionally, life-cycle testing (Figures S1A and S1B) showed that repeated delivery (100 injections) of cell culture media did not affect the implant pore size when compared with unused implants. Overall, delivering eNK cells and IL-15 via the therapeutic implant was as effective as standard 32G needle injection in terms of cell viability, cell concentration, and IL-15 delivery.

The force required to inject a therapy through the implant and to sample fluid using the implant was investigated. An injection force of 0.68 ± 0.17 N and a sampling force of 2.42 ± 0.13 N were measured (Figure 1L). The required injection and sampling forces are considerably less than the average maximum thumb force (100.62 N for women and 136.71 N for men^40^) and the average maximum pinch forces (50 N for women and 70 N for men^41^), indicating that this implant could easily be replenished by a single clinician or health care professional. Finally, the burst pressure of non-porous implants was investigated; there was no significant difference in implants that were irradiated when compared with non-irradiated implants (Figure S1C).

### Delivery of therapy analog through the peritoneal implant in an ovarian cancer model

**Figure 2.**
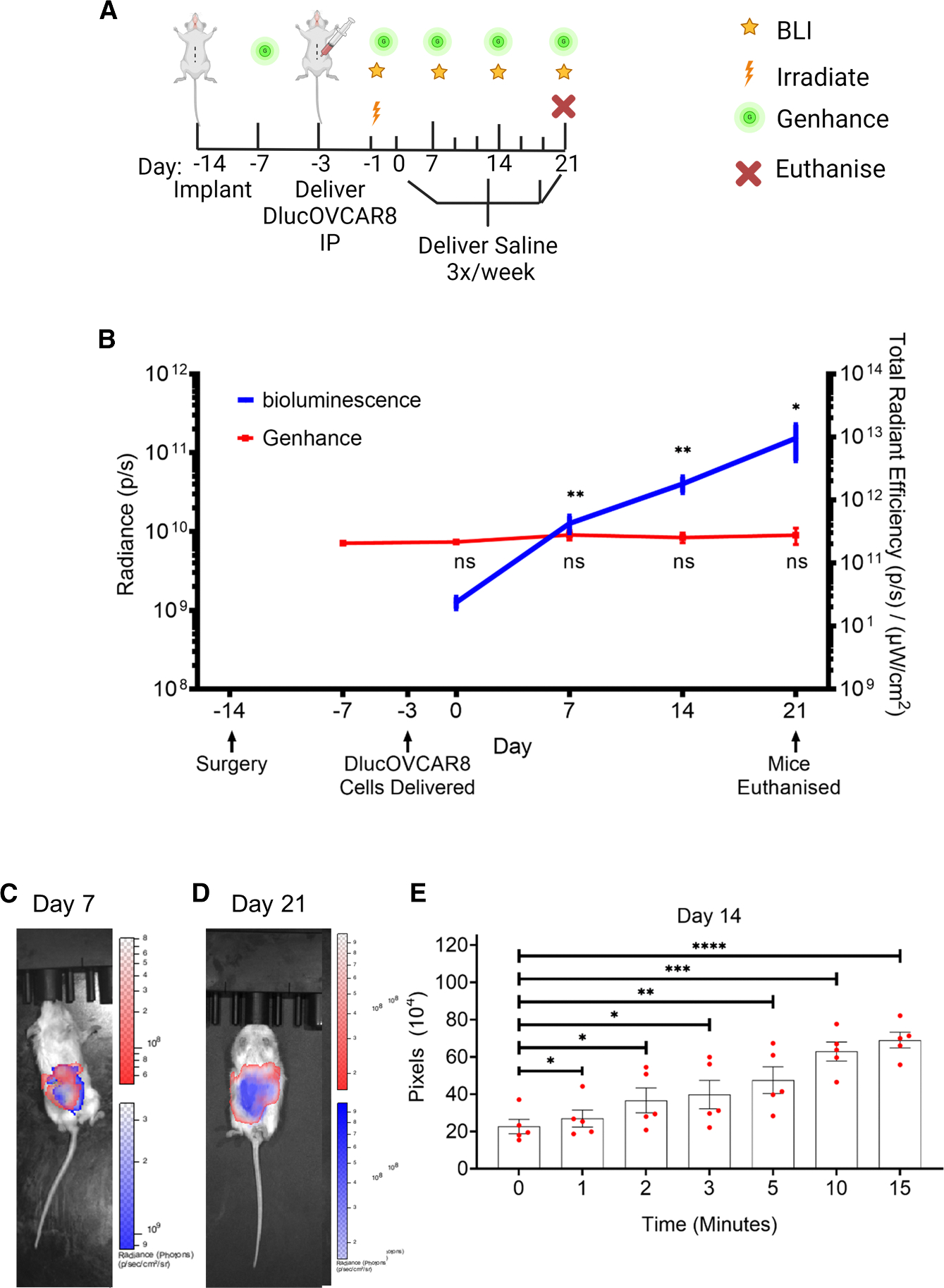
Implant can maintain consistent delivery of a therapy analog in the presence of increasing tumor burden (A) Preclinical study timeline used to evaluate delivery of therapy analog (Genhance) to i.p. space in D-luc+OVCAR-8 human ovarian NSG mouse cancer model (*n* = 5 mice). (B) Bioluminescence (blue line) shows quantified D-luc+OVCAR-8 tumor burden, while Genhance (therapy analog, red line) shows quantified therapy diffusion in the peritoneal cavity in mice, over 5 weeks. (C and D) Representative image of mice on days (C) 7 and (D) 21 with tumor burden (blue) overlaid with Genhance diffusional area (red) after 5 min. (E) The number of pixels detected for Genhance following delivery via the implant was quantified over 15 min. BLI, bioluminescence imaging; i.p, intraperitoneal; D-luc+OVCAR-8, human ovarian cancer cell line OVCAR-8 transfected with D-luciferase. Data are presented as mean ± standard error of mean. ns = *p* > 0.05, *p < 0.05, **p < 0.01, ***p < 0.001, ****p < 0.0001.

The feasibility of the implant to deliver a therapy analog in an ovarian cancer mouse model was evaluated (Figure 2A). We envisage that this implant would be surgically placed within the peritoneal cavity at the time of cytoreductive surgery. Although the goal of cytoreductive surgery is to achieve complete disease removal, many patients will have residual disease (<1 cm), which is a critical target for further therapeutic intervention. Our implant is intended for early deployment in the treatment course to address this residual disease and enable early intervention in the case of recurrent disease. The animal model used in this study was designed to reflect this clinical timeline, supporting its potential for clinical translation as a strategy to enhance local drug delivery and improve patient outcomes. The implant was surgically placed in the peritoneal cavity of four NSG mice on day −14. On day −3 (11 days post-surgery), 1 × 10^5^ D-luc+OVCAR-8 cells were administered through a 200 μL i.p. injection, before animals were exposed to low-dose total body irradiation (TBI) at a dose of 200 cGy on day −1 to provide a controlled tumor growth rate. D-luciferin (200 μL) was delivered via i.p. injection to monitor tumor burden weekly through bioluminescence imaging (BLI) of the D-luc+OVCAR-8 cells using an IVIS Spectrum Imaging System. Fluorescence imaging using the imaging substrate IVISense Acute Vascular 680 Fluorescent Probe (also known as Genhance)^42–44^ was used to validate rapid, targeted delivery through the implant into the i.p. space. Genhance was delivered through the implant via the transcutaneous port on days −7, 0, 7, 14, and 21. Additionally, to replicate a therapeutic regime, 200 μL of saline was delivered through the implant via the transcutaneous port three times/week for 3 weeks from day 0.

The tumor burden, as indicated by the radiant flux (photons/s) measured through bioluminescence imaging (blue line), steadily increased from day 0 to day 21. Despite this rise in tumor burden, delivery of Genhance through the implant remained consistent (red line) over the 21-day period (Figure 2B). There was a significant increase in tumor burden at days 7 (1.255 ± 5.19 × 10^10^ p/s), 14 (4.028 ± 1.56 × 10^10^ p/s), and 21 (15.26 ± 7.34 × 10^10^ p/s) compared with bioluminescence baseline day 0 (1.26 ± 0.361 × 10^9^ p/s). There was no significant change in the total radiant efficiency of Genhance psμWcm2 from days −7 (before delivery of D-luc+OVCAR-8 cells, 2.06 ± 0.166 × 10^11^), 0 (2.16 ± 0.475 × 10^11^), 7 (2.78 ± 0.921 × 10^11^), 14 (2.53 ± 0.993 × 10^11^), and 21 (2.77 ± 0.802 × 10^11^). Representative images depicting tumor burden (blue) and Genhance diffusion (red) within the i.p. space are shown for mice on days 7 (Figure 2C) and 21 (Figure 2D). On days 7, 14, and 21 post-D-luc+OVCAR-8 cell delivery, fluorescence images taken 0, 1, 2, 3, 5, 10, and 15 min post-delivery of Genhance through the implant were analyzed to evaluate temporal diffusion. On day 14, Genhance delivery via the implant, as quantified fluorescent pixels, increased significantly from baseline at 0 (22.67 ± 7.64) to 1 (26.97 ± 9.15), 2 (36.71 ± 13.38), 3 (39.80 ± 15.23), 5 (47.55 ± 14.41), 10 (61.90 ± 10.13), and 15 (69.06 ± 8.50) min post-delivery (Figure 2E). This trend was consistent on days 7 (Figure S2C) and 21 (Figure S2D). These data indicate that we can maintain consistent delivery to the i.p. space via the implant in an ovarian cancer setting. No implant-related complications were observed in any of the studies reported in this manuscript; see Table S2 for a full breakdown.

### Functionality of the peritoneal implant for 70 days in an ovarian cancer model

**Figure 3.**
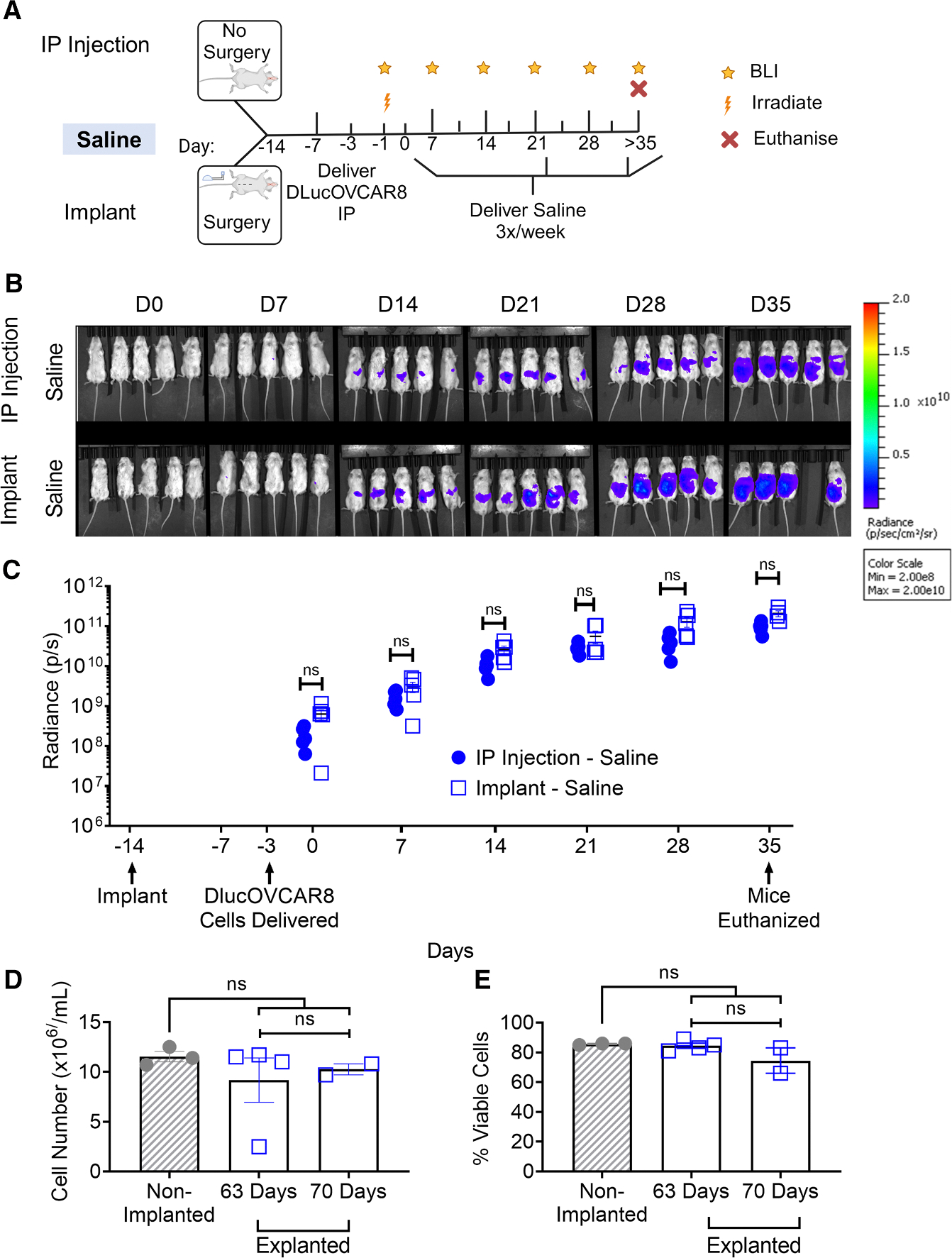
Implant does not alter tumor burden and remains functional up to 70 days (A) Preclinical study timeline used to evaluate D-luc+OVCAR-8 i.p. tumor growth in NSG mice with and without the implant. (B) *In vivo* images of tumor growth with and without implant over 35 days (min: 2 × 10^8^ and max: 2 × 10^10^). (C) Tumor burden was quantified after the therapeutic regimen (saline) was delivered via implant compared with i.p. injection over 35 days. (D and E) Implants were explanted at days 63 (*n* = 4) and 70 (*n* = 2), and peripheral blood mononuclear cells were delivered through explanted reservoirs and non-implanted reservoirs (*n* = 3). (D) Viability and (E) cell number delivered were quantified. BLI, bioluminescence imaging; i.p, intraperitoneal; D-luc+OVCAR-8, human ovarian cancer cell line OVCAR-8 transfected with D-luciferase. Data are presented as mean ± standard error of mean. ns = *p* > 0.05.

We investigated whether our peritoneal implant altered D-luc+OVCAR-8 cell growth in the peritoneal cavity of NSG mice (Figure 3A). The implant was surgically placed in the peritoneal cavity of 11 NSG mice on day −14. An additional 5 mice did not receive the implant. As in the previous section, 1 × 10^5^ D-luc+OVCAR-8 cells were administered on day −3, and animals were irradiated on day −1; tumor burden was monitored weekly through BLI (Figure 3B). To replicate a therapeutic regime, 200 μL of saline was delivered three times/week for 5 weeks (day 35) through our implant and, in this case, compared with i.p. injection via a 32G needle. There was no significant difference in BLI radiance (p/s) between mice that received the implant and those that did not, at any time point (Figure 3C; Table S3). Mice that received the implant exhibited a higher baseline BLI reading (6.32 × 10^8^ ± 3.62 × 10^8^) compared with the i.p. injection group (1.85 × 10^8^ ± 9.26 × 10^7^), though this was not statistically significant. This difference is evident from day 0, suggesting that surgery may alter tumor cell engraftment. To directly examine the tumor progression rate for i.p. injection vs. implant groups, each reading (mouse) was normalized to its day 0 BLI value (Figure S3) and calculated for each time point, and there was no significant difference at any time point. This indicates that the implant did not exacerbate tumor growth relative to controls.

To explore the lifespan of the implant, six mice were retained until they reached humane endpoints at days 63 (4 mice) and 70 (2 mice). Post-euthanasia, implants were explanted, and 10 × 10^6^ peripheral blood mononuclear cells (PBMCs) were delivered *in vitro* through the explanted porous reservoirs and compared with fresh non-implanted reservoirs. There were no significant differences in PBMC viability post-delivery in all groups (11.53 ± 0.91 × 10^6^ vs. 9.17 ± 4.47 × 10^6^ and 10.25 ± 0.78 × 10^6^ cells/mL, Figure 3D). Similarly, there was no significant difference in the number of cells delivered through non-implanted reservoirs and those that had been implanted for 63 or 70 days (85.67% ± 0.58% vs. 84.50% ± 3.42% and 74.50% ± 12.02%, Figure 3E). Therefore, the implant can effectively deliver cells up to 70 days post-implantation.

### Efficacy of implant compared to i.p. injection

**Figure 4.**
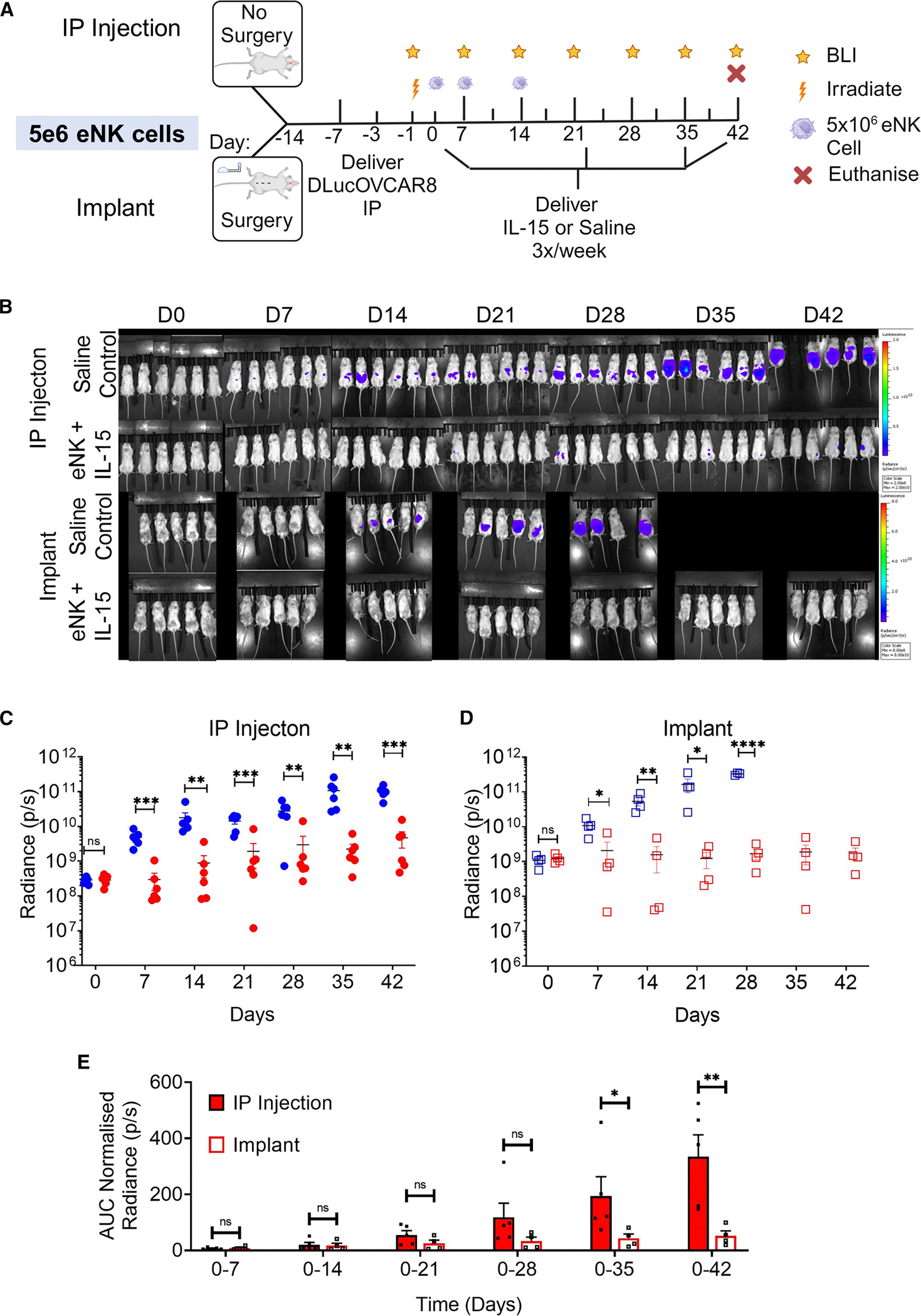
Therapy delivery via implant controls tumor burden better than gold-standard i.p. injection 35 and 42 days after initiating treatment (A) Preclinical study timeline used to evaluate eNK cell therapies with IL-15 through the implant or i.p. injection. (B) *In vivo* images of tumor growth with and without implant over 42 days (min: 8 × 10^8^ and max: 8 × 10^10^). (C and D) Tumor burden was quantified after delivery of our therapeutic regimen compared with saline (control), via either (C) i.p. injection or (D) delivery through the therapeutic implant over 42 days. Only animals with confirmed tumor engraftment at day 0 were included in quantitative analysis (*n* = 6 mice/i.p. injection, *n* = 4 mice/implant). (E) To directly compare the delivery of eNK cells + IL-15 via our implant or via i.p. injection at each time point, we normalized each reading to its day 0 BLI value and calculated the AUC for day 0 to each time point. BLI, bioluminescence imaging; i.p., intraperitoneal; D-luc+OVCAR-8, human ovarian cancer cell line OVCAR-8 transfected with D-luciferase; eNK, expanded natural killer cells; IL-15, interleukin-15; AUC, area under the curve. The therapeutic regimen is shown in red. Saline (control) is shown in blue. Data are presented as mean ± standard error of mean. ns = *p* > 0.05, *p < 0.05, **p < 0.01, ***p < 0.001, ****p < 0.0001.

Therapeutic delivery through the implant in the context of controlling ovarian cancer tumor burden was evaluated. The implant was surgically placed in the peritoneal cavity of 10 NSG mice on day −14. An additional 12 NSG mice did not receive the implant and were allocated to a group for therapy via i.p. injection (32G needle). 1 × 10^5^ D-luc+OVCAR-8 cells were administered through a 200 μL i.p. injection on day −3 before animals received TBI on day −1. Tumor burden was monitored weekly via BLI. Four experimental groups were investigated: i.p. injection of saline, implant delivery of saline, i.p. injection of eNK + IL-15, and implant delivery of eNK + IL-15. Animals in each of the saline control groups received 200 μL of saline via i.p. injection (32G needle) or through the implant via the transcutaneous port three times/week from day 0. Animals in each of the treatment groups received 5 × 10^6^ eNK cells via i.p. injection (32G needle) or through the implant once per week for 3 weeks (at days 0, 7, and 14) and 1 ng IL-15 in 200 μL (4.9 ng/mL) via the same route three times/week from day 0 (Figure 4A).

Luminescence images for all animals are shown in Figure 4B. At each of the time points measured, after day 0, treatment with eNK + IL-15 reduced the total radiant flux of the D-luc+OVCAR-8 cells relative to saline controls when delivered through i.p. injection (Figure 4C; Tables S4 and S5). The same trend was observed with implant-delivered therapy (Figure 4D; Tables S6 and S7). These data show that the therapeutic regimen impacts tumor load in these mice.

The survival curve of mice receiving treatment via i.p. injection showed no survival benefit compared with saline controls (Figure S4A). However, the survival curve of mice that received treatment via the implant demonstrated a significant survival benefit for mice receiving eNK + IL-15 compared to saline control (Figure S4B, **p = 0.0067 as determined by log rank [Mantel-Cox] test). It was identified that surgery moderately accelerates tumor engraftment, resulting in mice that received the implant exhibiting a higher baseline BLI reading (1.15 × 10^9^ ± 3.28 × 10^8^) compared to the i.p. injection group (2.89 × 10^8^ ± 7.20 × 10^7^). The higher initial BLI in the implant group could explain the earlier mortality in the mice that received saline through the implant between days 28 and 35, as the implant group reached a BLI of 3.28 × 10^11^ by week 28, compared with 1 × 10^10^ for i.p. injection mice at the same time point.

To directly compare the delivery of eNK cells + IL-15 via our implant with that through i.p. injection at each time point, we normalized each reading to its day 0 BLI value (to normalize for variability in tumor establishment and growth kinetics across groups), and the area under the curve (AUC) was quantified from day 0 to each time point (Figure 4E). The AUC is commonly used to measure the cumulative effect of a treatment on tumor burden over time, helping to compare different therapies’ effectiveness. Up until day 28, there was no significant difference in the tumor control when we compared therapy delivery through the implant to i.p. injection (Figure 4E; Tables S6 and S7). However, from day 0 to days 35 and 42, we show a significant improvement in controlling tumor burden when our therapeutic regimen was delivered through the implant compared with i.p. injection (days 0–35: 193.54 ± 138.18 vs. 42.99 ± 24.47, *p* < 0.05, and days 0–42: 334.58 ± 155.56 vs. 52.16 ± 29.98, *p* < 0.01).

To evaluate the ex vivo function of an implant used to deliver cells and cytokines in vivo (unlike in Figures 3D and 3E, where sterile saline alone was delivered in vivo), the implants were explanted, and 10 × 10^6^ eNK cells were delivered through the porous reservoirs *in vitro* and compared with fresh, non-implanted reservoirs. Minor differences in cell viability (62.67% ± 2.06%, vs. 56.00% ± 5.35% and 60.33% ± 1.89%, Figure S4C) or cell number (11.53 ± 0.91 × 10^6^ vs. 9.17 ± 4.47 × 10^6^ and 10.25 ± 0.78 × 10^6^, Figure S4C) were observed when delivered through non-implanted, explanted reservoirs from the saline group and explanted reservoirs from the eNK + IL-15 group. The viability and number of eNK cells delivered through the explanted therapeutic implants were comparable with those delivered through fresh, non-implanted implants, indicating consistent implant performance even after three therapeutic cell doses and weekly cytokine dosing for 42 days. Collectively, these results indicate that delivery via the implant achieved comparable early responses and superior long-term tumor control relative to i.p. injection, highlighting its potential to enable repeated, localized immunotherapy delivery in a clinically translatable manner.

### Minimally invasive sampling of tumor and immune cells from i.p. space

**Figure 5.**
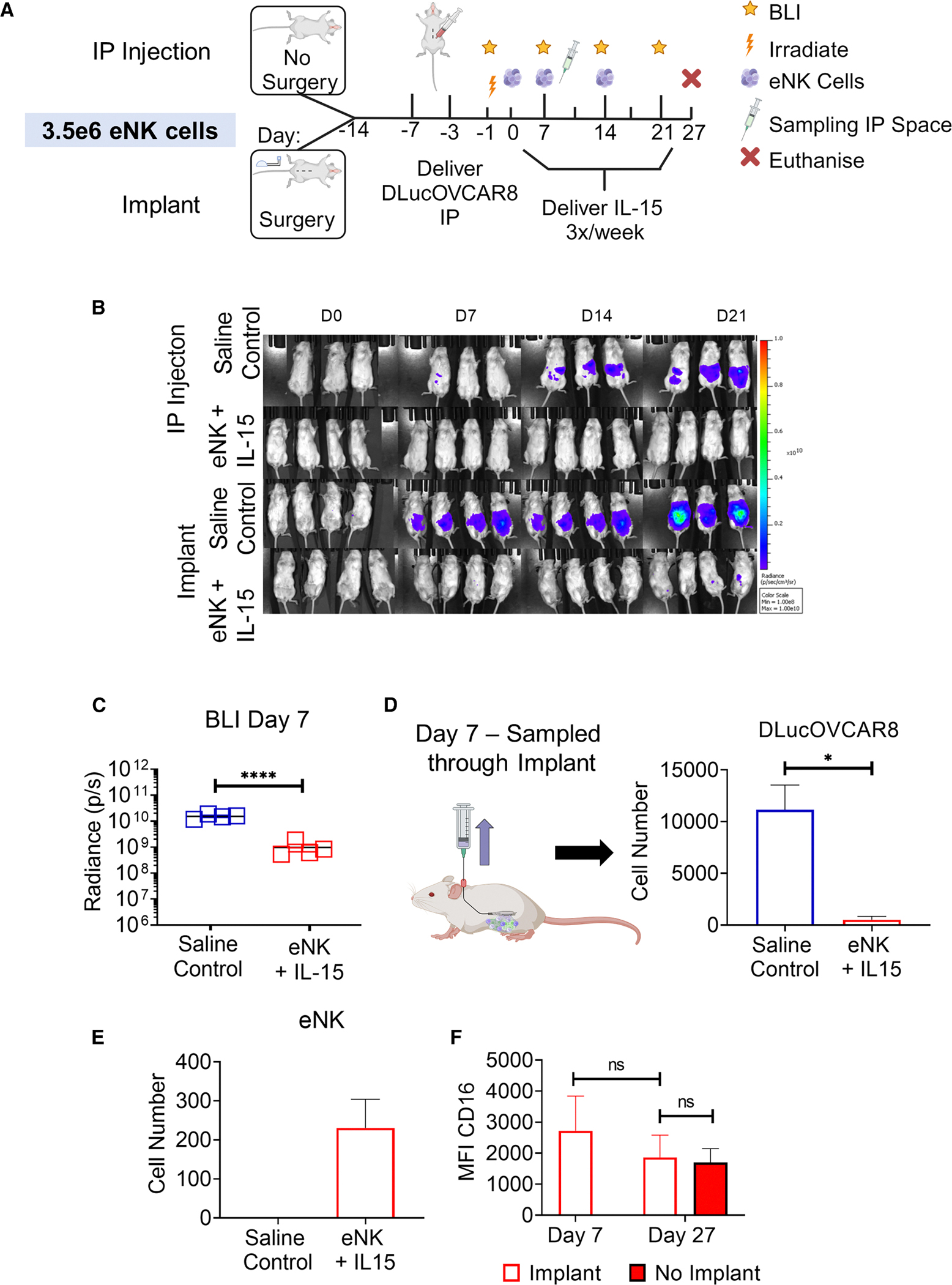
Implant can sample peritoneal fluid to monitor the peritoneum and tumor microenvironment (A) Preclinical study timeline used to evaluate cell sampling through the implant (*n* = 4 mice/group). (B) *In vivo* images of tumor growth with and without implant over 21 days (min: 1 × 10^8^ and max: 1 × 10^10^). (C) Tumor burden was quantified for saline control and eNK + IL-15 implant groups on day 7. Negative pressure was applied to the transcutaneous port to allow peritoneal fluid to be minimally invasively collected on day 7. (D and E) The numbers of (D) D-luc+OVCAR-8 and (E) eNK cells in the peritoneal fluid on day 7 were quantified. (F) eNK cell function was identified through MFI for CD16, a marker associated with ADCC, on cells sampled on day 7 and i.p. lavage samples on day 27. MFI, median fluorescence intensity; BLI, bioluminescence imaging; i.p., intraperitoneal; D-luc+OVCAR-8, human ovarian cancer cell line OVCAR-8 transfected with D-luciferase; eNK, expanded natural killer cells; IL-15, interleukin-15. The therapeutic regimen is shown in red. The saline (control) is shown in blue. Data are presented as mean ± standard error of mean. ns = *p* > 0.05, *p < 0.05, ****p < 0.0001.

The feasibility of the implant to minimally invasively sample cells from peritoneal fluid was assessed (Figure 5A). In this case, a lower dose of 3.5 × 10^6^ eNK cells was used, and the study duration was shortened; mice were euthanized on day 27. As before, the implant was surgically placed in the peritoneal cavity of 8 NSG mice on day −14, with an additional 10 NSG mice serving as controls. 1 × 10^5^ D-luc+OVCAR-8 cells were administered through a 200 μL i.p. injection on day −3, followed by TBI on day −1. Tumor burden was monitored weekly via BLI. Four experimental groups were investigated: i.p. injection of saline, implant delivery of saline, i.p. injection of low-dose eNK + IL-15, and implant delivery of low-dose eNK + IL-15. In the saline control groups, 200 μL of saline was administered via i.p. injection (32G needle) or via the transcutaneous port three times/week from day 0. eNK cells (3.5 × 10^6^) were delivered via i.p. injection or through the implant once per week for 3 weeks from day 0, along with 1 ng IL-15 in 200 μL administered three times/week from day 0. Peritoneal fluid was sampled through the implant on day 7 while the animals were on study (*n* = 2/group). 1,000 μL of sterile saline was delivered to the i.p. space via the implant, and 800 μL was aspirated back through the implant via the transcutaneous port. Following euthanasia on day 27, i.p. lavage was performed; 7 mL of sterile PBS was delivered to and aspirated from the i.p. space via pipette. Collected cells from the sampled peritoneal fluid and i.p. lavage were stained to identify tumor and NK cells.

To validate our model with a lower eNK cell dose, we investigated tumor growth over time. Similar to the higher eNK cell dose (Figure 4), radiance (p/s) increased in the saline controls when delivered via our implant or through i.p. injection, while no increase in BLI in the groups treated with eNK + IL-15 was observed (Figures 5B and 5C). As before, we normalized each reading to its day 0 BLI value, and the AUC was quantified. Consistent with Figure 4, eNK + IL-15 controlled tumor burden significantly better than saline control from day 7 to day 21, regardless of delivery route (Figure S5; Table S8).

To demonstrate proof of concept, peritoneal fluid was sampled through our implant via the transcutaneous port on day 7, which enabled direct monitoring of tumor and immune cell presence in the peritoneum. D-luc+OVCAR-8 cells and eNK cells (human [h]CD56+hCD3−) were identified in the peritoneal fluid collected via our implant on day 7 (Figures 5D and 5E). A significant difference was found in the number of tumor cells in the peritoneal fluid between the saline control and eNK + IL-15 group (11,155 ± 1,196 vs. 733 ± 119, *p* < 0.05 Figure 5D). This finding aligns with our BLI results, which showed a significant increase in tumor burden for saline control compared with eNK + IL-15 (*p* < 0.0001 Figure 5C). hCD56 cells were detected in the peritoneal fluid on day 7 and quantified for the saline control and eNK + IL-15 (0 and 231 ± 74, Figure 5E). These data show that the implant can be used to isolate, detect, and quantify cancer and immune cells from peritoneal fluid.

We analyzed eNK cell functional maturity by measuring mean fluorescence intensity (MFI) for CD16. CD16 is a marker associated with antibody-dependent cell-mediated cytotoxicity (ADCC) and appears on NK cells at later stages of development. It was assessed on eNK (hCD56+CD3−) cells (Figure 5F) that were collected via sampling on day 7 or via peritoneal lavage post-euthanasia on day 27. No significant difference in CD16 MFI was observed on eNK cells on day 7 vs. day 27 (2,725 ± 1,115 vs. 1,864.5 ± 1,017.1) in animals that received the implant. There was also no significant difference in CD16 expression on eNK cells on day 27 from animals that received the implant vs. animals that had no implant and received eNK via i.p. injection (1,864.5 ± 1,017.1 vs. 1,701.3 ± 632.0). This highlights that the implant does not adversely affect CD16 levels on NK cells, a key marker of ADCC and cytotoxicity.

Overall, sampling peritoneal fluid via the implant on day 7 successfully enabled direct access to the i.p. space. Importantly, we identified both D-luc+OVCAR-8 cancer cells and therapeutic eNK cells in the collected fluid, demonstrating the potential to facilitate a detailed cellular analysis of both tumor and immune cells.

## DISCUSSION

The implantable platform technology presented here combines medical implant design and cell/drug delivery expertise to provide localized, repeated therapeutic delivery and sampling in the peritoneal cavity. This range of applications is unaddressed by existing technologies, despite a need for innovative and sophisticated approaches in peritoneal malignancies and other diseases that present in the peritoneal cavity.

The combination of eNK cell therapy with IL-15 exhibited potent cytolytic effects against human ovarian cancer cells *in vitro*, consistent with prior literature,^45,46^ and was chosen as the therapy to evaluate the performance of the implant in a xenograft model of ovarian cancer. We have shown that the implant can support the delivery of cell therapies (including eNK cells and PBMCs) *in vitro* and *ex vivo*, with no significant effect on the number or viability of cells delivered through the implant compared with injection through a 32G needle. When implanted in the peritoneal cavity of a human ovarian cancer mouse model, there was consistent delivery of a therapy analog (Genhance) for 35 days, despite an increase in tumor burden. We also have shown that the implant itself did not exacerbate tumor growth, with no significant difference in the normalized D-luc+OVCAR-8 luminescence between animals with and without implants for up to 35 days. After 35 and 42 days of eNK + IL-15 therapy, animals that received treatment through the implant had significantly reduced tumor burden compared with i.p.-injection-treated animals. *Ex vivo* analysis showed that cell therapy (PBMCs) could be delivered through the porous membrane of the therapeutic reservoir for up to 70 days. Taken together, these results highlight the potential of our implant as a more targeted method for sustained, long-term therapeutic delivery in ovarian cancer. This dual-purpose implant also facilitates peritoneal fluid sampling for longitudinal tumor and immune cell monitoring. For instance, our sampling data indicate an increase in cancer cells in the saline-treated group, which correlates with increased tumor burden as measured by BLI. Additionally, the capacity to track NK cell number and functionality in real time offers valuable insights into cell persistence and efficacy. This feature would allow clinicians to monitor disease progression instantaneously and adjust treatment accordingly, a significant and novel advancement in the field.

In clinical practice, localized delivery of therapeutics to the i.p. space is currently facilitated by repurposed catheters, such as those designed for hemodialysis.^21–24^ There is an unmet clinical need for a purpose-built platform for the localized and repeated delivery of therapy to the i.p. space. Coon et al. investigated nitinol thin films (TFNs) for CAR-T cell therapy in an OVCAR-3 human ovarian cancer NSG mouse model.^3^ Surgically implanted TFN micromeshes loaded with 10 × 10^6^ CAR-T cells significantly increased the median survival of cancer-bearing mice relative to those treated with i.p. and i.v. injection of the same therapy. Smith et al.^47^ used an implanted alginate biopolymer scaffold to deliver CAR-T therapy to treat inoperable pancreatic cancer in C57BL/6 mice. This platform improved the survival of cancer-bearing mice compared with those treated with localized injection of the same therapy. Using another approach for therapy release, Nash et al. developed surgically implanted “cytokine factories” using polymer-encapsulated human retinal-pigmented epithelial cells engineered to produce IL-2.^48^ In a C57BL/6 mouse ovarian cancer model (ID8 cells), this platform achieved >90% tumor reduction and significantly extended survival compared with animals treated with IL-2 injection. Ye et al.^2^ and Tanenbaum et al.^49^ developed a therapeutic reservoir implanted in the i.p. space of BALB/c nude mice to provide diffusion-controlled release of cisplatin for the treatment of ovarian cancer.^2,49^ Their implant performed on par with i.p. cisplatin injection and significantly reduced tumor burden relative to untreated controls but resulted in significantly less toxicity (quantified by white blood cell counts). However, the authors note challenges with the *in vivo* release profile, with only 20% of the initial cisplatin payload being released over 42 days. These studies demonstrate the potential of indwelling therapy release platforms to improve cancer treatment, even when compared with localized therapy delivery via i.p. injection. However, all of these approaches are limited to a pre-loaded therapeutic, with no ability to longitudinally adjust the dose, frequency, or type of therapy after implantation. Furthermore, these approaches do not provide any mechanism for real-time monitoring of therapeutic efficacy.

In the current study, the implant size and shape were designed for surgical placement in a mouse model of ovarian cancer. Other cancers, such as gastric, colorectal, pancreatic, and liver,^50^ within the peritoneum may also benefit from localized therapeutic delivery. Similar to ovarian cancer, a systematic review of the literature found that many of these patients can only avail i.p. chemotherapy intra-operatively, with no device available for long-term use.^51^ This highlights a potential clinical benefit for our implantable platform technology beyond ovarian cancer. Additionally, previous work by our group^52^ described an implant scaled up for i.p. delivery for porcine and human studies. In that work, we devised a percutaneous, over-the-wire approach to position the implant in the peritoneal space based on the Seldinger technique. The implant was then secured minimally invasively using a porous bioadhesive reservoir to deliver medical-grade cyanoacrylate. While implantation in the current manuscript is intended to coincide with cytoreductive surgery, the flexible implant can also feature a percutaneous, over-the-wire approach that enables implantation at later stages. This method, compatible with interventional radiology techniques, allows for precise positioning and potential re-implantation if necessary, making it adaptable for other clinical scenarios. This approach could offer an alternative treatment delivery modality for inoperable cancers, such as pancreatic cancer,^53^ or chronic inflammatory diseases, such as Crohn’s disease,^54,55^ conditions that could both benefit from novel experimental biologics or immune-based therapies^56^; however, further studies would be required.

More than 90% of patients with advanced ovarian cancer present with ascites,^34^ an accumulation of fluid in the abdominal cavity that contains a range of tumor and non-tumor cells, as well as cell-free DNA and signaling molecules that influence cell behavior.^34^ Ascites is observed in 20%–50% of patients with peritoneal malignancies, such as gastric, colorectal, pancreatic, and liver.^50^ Collecting samples of peritoneal cells during treatment could provide important information to adjust the therapy as needed in real time. Ascites contributes to patient morbidity and mortality by facilitating metastasis and chemoresistance.^34^ Clinically, it can lead to discomfort, abdominal distension and pressure, dyspnea, bloating, pelvic pain, and bowel or bladder dysfunction.^57^ The most commonly used device for ascites removal is the PleurX Catheter System by Teleflex Medical, which is a silicone catheter with a one-way valve,^58^ which was originally designed for draining fluid from the pleural region.^58,59^ Due to the recurrent nature of ascites, the median of symptom relief is 10.4 days.^59^ Once implanted, treatment with a PleurX catheter can be required for months or even years,^59^ with the catheter remaining *in situ* unless complications arise. These are often implanted by interventional radiologists when patients have relapsed disease^59^ and stay in for weeks to months, depending on the patient’s needs and the catheter condition.^58–60^ While this can alleviate symptoms by reducing peritoneal cavity volume, no published literature specifically describes it being used for cell sampling. Instead, analysis of ascites usually occurs during cytoreductive surgery,^58,59^ limiting the opportunity for ongoing monitoring.^60^ In addition, ascites analysis in the preclinical evaluation of new ovarian cancer therapies tends to be a one-time procedure,^61^ hindering longitudinal and real-time monitoring of disease progression, the tumor microenvironment, and therapeutic response.^34^ In contrast, our implant offers the capability for real-time sampling of peritoneal cells, enabling the assessment of cellular and molecular components of the peritoneal environment, while also allowing for ascites drainage as required. Here, we analyzed the type, number, and CD16 expression of cells sampled from the i.p. space at one time point, demonstrating proof of concept. However, future work could include a more in-depth analysis of immune cell populations at various time points along the disease trajectory to facilitate more effective treatment strategies, as well as in-depth genetic analysis of free-floating tumor cells to evaluate disease progression.

## Conclusion and outlook

There are some limitations with the current study. Here, we evaluated our peritoneal implant in an immunocompromised murine model. This model supports human cell engraftment,^27,31,32,62^ allowing for evaluation of therapies against human cancer cells. However, the mouse model used is deficient in T, B, and NK cells, with compromised macrophages and dendritic cells and a reduced complement system.^63^ Therefore, it does not fully replicate the complexity and heterogeneity of human ovarian cancers or immune interactions,^64^ even though NK cells are introduced in the therapeutic groups. Nevertheless, it remains the standard murine model for assessing human cell immunotherapy function in preclinical studies.^31,32,45^ Future studies should utilize the syngeneic murine ID8 ovarian cancer model to test the implant in immune-competent models such as C57BL/6 mice,^44,47^ which are commonly used to study immune responses and cancer,^45^ as well as device/host interactions.^44,47^ In addition, the performance of the implant for localized delivery of alternative therapeutic regimes, such as chemotherapy,^65^ PARP inhibitors,^66^ tri-specific killer engagers (TriKEs),^67^ and immune checkpoint inhibitors,^68^ could be explored in future work. While this study was performed in mice, our group has previously demonstrated the feasibility of a similar platform in an acute porcine model, confirming i.p. access, anchoring, and function.^52^ These data support the adaptability of the technology to larger animals and provide a foundation for future chronic large-animal studies to assess long-term safety and performance in a more clinically relevant setting. A direct head-to-head comparison with existing i.p. catheter systems was not feasible, as no equivalent systems exist for use in murine models. Nonetheless, across all *in vivo* studies reported here (*n* = 33 mice), there were zero implant-related complications (0%), including infections, perforations, dislodgements, blockages, or port access problems (Table S2), and implants remained functional upon explantation (Figures 3D, 3E, S4D, and S4E). While this proof-of-concept study highlights the potential of a specifically designed i.p. delivery implant, future studies in large-animal models will compare this implant against existing clinically used catheters. Finally, this present study focused on a membrane with 100-μm pores to enable the delivery of cellular components. Future work could investigate how variations in porosity influence release kinetics and host response to tune therapeutic exposure within the peritoneal cavity. Importantly, unlike previously reported pre-loaded sustained-released implants,^2,3,49^ our replenishable platform offers the flexibility to adjust dosing profiles and therapeutic composition post-implantation, enabling more precise and adaptive treatment control.

In this study, we note that mice that received the implant via surgical placement tended to start at a (non-significantly) higher BLI in each study (initial signal approximately half a magnitude higher, roughly 8.5 × 10^6^ vs. 9 × 10^6^). Given that the initial differential is present from day 0, it is unlikely to be a result of the implant but rather is likely from the surgery, facilitating enhanced tumor engraftment. One potential explanation is that monocytes, typically involved in allogeneic cell rejection, may have been redirected to tissue repair functions in response to surgical injury.^69^ To minimize variability in future studies, performing sham surgeries on the control mice could help to standardize tumor engraftment conditions. While this study focuses on eNK cells in combination with IL-15 as a therapy to treat ovarian cancer, the versatile nature of the implant means that it could be used to deliver a wide range of cell- or biotherapeutic-based therapies.

The implant presented here is part of our efforts to use advanced delivery and monitoring technologies to tackle well-known healthcare challenges. By leveraging insights from our previous work,^42,44,52,70,71^ future iterations of this implant could include a range of features, including sensors and mechanisms to overcome any blockages caused by long-term implantation. Indeed, uneven drug exposure between quadrants in the peritoneum is a well-recognized limitation of current catheter-based approaches.^72^ As outlined in Figure 1, our platform is highly adaptable and could incorporate one or multiple reservoirs positioned across peritoneal quadrants, allowing both uniform coverage and the option to target sites of highest disease burden even more specifically.

## METHODS

### Manufacture and benchtop testing of the peritoneal implant

Therapeutic reservoirs were manufactured from TPU polyether film (American Polyfilm) with 100-μm porous membranes and connected to Micro-Renethane catheter tubing (MRE025, Braintree Scientific, with an inner diameter of 0.305 mm, an outer diameter of 0.635 mm, and a length of 5 cm). The assembly was connected to a self-sealing transcutaneous access port (VABM1B/22, Instech Laboratories). The volume of the reservoir was 56 μL, and the dead volume in the system was 15 μL (total volume = 71 μL). The full manufacturing process is provided in the supplemental information (Note S1).

To measure injection force, a 22G blunt fill needle and a 1 mL syringe filled with water were connected to the catheter, and the reservoir was submerged. A Zwick/Roell Biaxial tester compressed the syringe at a rate of 57 mm/min for 1 min, to deliver a total volume of 100 μL through the reservoir. This was repeated in triplicate, with five technical replicates per reservoir.

To assess pore size and spacing after repeated injections, light microscopy (BX Series Upright Metallurgical Microscopes, BX53M) was used before and after 100 injections of 69 μL of RPI 1640 medium via syringe pump at 0.5 mL/min.

For mechanical integrity testing, non-porous reservoirs were irradiated at 200 cGy before being connected to a 30G blunt fill needle and a 1-mL syringe. A syringe pump (NewEra AL-300) was used to inject water into the implant at 1 mL/min until burst. Burst pressure was recorded with a sensor (STORKSOLUTIONS, UPS-HSR-B1000G) and compared to non-irritated reservoirs (*n* = 6/group).

### *In vitro* studies

eNK cells were prepared according to Somanchi et al.^36^ The full expansion process is provided in the supplemental information (Note S2). OVCAR-8 human ovarian cancer cells were obtained in 2017 from the DTP/DCTD Tumor Repository sponsored by the Biological Testing Branch, Developmental Therapeutics Program, NCI, NIH (Frederick, MD; RRID: CVCL_1629). They were confirmed to be mycoplasma free when used and confirmed to be OVCAR8s by STR testing.

The cell number and viability of eNK cells from three donors were compared post-delivery through implant or a 32G needle. 200 μL of cell suspension (0.3 × 10^6^ cells) was delivered, and cell number and cell viability were assessed using a Celica MX Nexcelom cell counter (triplicate, three technical replicates).

IL-15 (0.15 mg/mL) concentration post-delivery through the implant was assessed using an A280 machine reader (triplicate, three technical replicates).

To assess eNK cell cytotoxicity, 20 × 10^5^ D-luc+OVCAR-8 human ovarian cancer cells were plated in an xCELLigence 96-well plate (RTCA MP, Agilent). After 24 h, eNK cells at a 2:1 effector:target ratio in combination with 10 nM IL-15 were added. The cell index was measured at 0.5, 1, and 4 h, and the percentage of cytolysis was calculated using xCelligence software (five donors, three technical replicates).

### *In vivo* studies

Mouse studies were carried out after approval (protocol 2207–40255A) from the Institutional Animal Care and Use Committee (IACUC) at the University of Minnesota and in compliance with their guidelines. All studies were performed in female NSG mice weighing 19–21 g. Mice received the implant surgically in the peritoneal cavity on day −14. i.p. injection controls did not receive implants. On day −3, all mice received 1 × 10^5^ D-luc+OVCAR-8 cells via 200 μL i.p. injection (32G needle). Animals received TBI at 200 cGy (X-Rad320) on day −1. Tumor burden was monitored weekly via BLI using an IVIS Spectrum Imaging System (PerkinElmer, Waltham, MA) after i.p. injection of 200 μL of D-luciferin (150 mg/kg, PerkinElmer). Tumor burden (p/s) was calculated, and mice were divided into the needed groups. This was performed under anesthesia. The full surgical process is provided in the supplemental information (Note S3; Figure S6). To ensure consistency in data analysis, only animals with confirmed tumor engraftment at day 0 were included in the quantitative analysis.

Collected cells from sampled peritoneal fluid and i.p. lavage were stained for murine (m)CD45 (BioLegend, 103149), hCD45 (BioLegend, 368523), CD56 (BioLegend, 362541), CD3 (BioLegend, 300449), and CD16 (BioLegend, 302017) markers. Mouse and human leukocytes were distinguished through mCD45 and hCD45 staining. NK cells were identified within the hCD45^+^ population using CD56^+^ and CD3^−^ markers, with CD16^+^ indicating NK cell functional maturity and potential to mediate ADCC GFP-tagged D-luc+OVCAR-8 cells were also identified during our analysis.

### Statistical analysis

Statistical analysis of data was performed using GraphPad Prism 8 software. All data were checked for normality prior to carrying out statistical analysis. All analysis was done using a multiple or *t* test (paired or unpaired) unless otherwise stated. Two-way ANOVA with a multiple comparisons test and Bonferroni post hoc test was used to assess Genhance diffusion over 0–15 min over 21 days. Two-way ANOVA with multiple comparisons and Bonferroni post hoc test were used to assess tumor burden growth between the implant and control groups. A log rank (Mantel-Cox) test was used to assess the survival curve of implant and i.p. delivery mice between control and eNK + IL-15 groups. Data are represented as the mean ± standard error of the mean. Significance: ns *p* > 0.05, *p < 0.05, **p < 0.01, ***p < 0.001, and ****p < 0.0001.

### RESOURCE AVAILABILITY

#### Lead contact

Requests for further information and resources should be directed to and will be fulfilled by the lead contact, Eimear B. Dolan (eimear.dolan@universityofgalway.ie).

#### Materials availability

Materials can be provided upon reasonable request to the corresponding authors following a materials transfer agreement. This study did not generate new unique reagents.

#### Data and code availability

The authors declare that all data supporting the findings of this study are available in the manuscript and supplemental information. Additional data can be provided upon reasonable request to the corresponding authors. 3D printing and design files have been made available. The images in Figures 1, 2, 3, 4, and 5 were made with BioRender. Science Suite dba BioRender (“BioRender”) has granted the following BioRender user, Aoibhín Sheedy (“user”), a BioRender academic publication license in accordance with BioRender’s terms of service and academic license terms (“license terms”).

## Supplementary Material

1

2

3

4

Supplemental information can be found online at https://doi.org/10.1016/j.device.2026.101050.

## THE BIGGER PICTURE

Local peritoneal delivery of therapies for abdominal cancers, such as ovarian cancer, has been shown to improve therapeutic outcomes. Despite this, clinical adoption of peritoneal delivery has been limited largely due to challenges associated with the medical devices that are available for this route of therapy administration. These issues cause over 30% of patients to discontinue lifesaving treatment. In this study, we designed a therapeutic implant for repeated delivery of cellular and biological therapies to the peritoneal cavity. Constructed from a flexible thermoplastic polymer, our implant facilitates intraperitoneal therapy delivery and permits peritoneal fluid collection to monitor the tumor microenvironment. This platform represents an avenue for personalized cancer treatments through repeated, controlled, and localized therapeutic administration and real-time monitoring.

## Highlights

- Implant enables repeated, localized delivery of therapies to the peritoneal cavity
- Delivery via implant controls tumor better than standard intraperitoneal injection
- Implant enables minimally invasive longitudinal sampling of peritoneal fluid
