# Supplementary material for: A replenishable peritoneal implant for localized delivery and peritoneal fluid sampling in ovarian cancer
Source: Device. Author manuscript; Available in PMC 2026 Apr 4. (PMC13048345; doi:10.1016/j.device.2026.101050)
Supplement: 1 [file NIHMS2154242-supplement-1.pdf]

DEVICE, Volume *4*

Supplemental information

A replenishable peritoneal implant for localized delivery and peritoneal fluid sampling in ovarian cancer

Aoibhin M. Sheedy, Mihir Shetty, Anna Weis, Laura E. Bendzick, Terran Stenger, Zhenya Ni, Philippa R. Kennedy, Jacob A. Myers, Niamh Ward, Lesley Trask, Hannah Prendeville, Joanne O'Dwyer, Michael O'Dwyer, Ellen T. Roche, Garry P. Duffy, Jeffrey S. Miller, Melissa A. Geller, Eimear B. Dolan, and Martin Felices

Table of Contents

Table S1: Currently repurposed catheters are being used to deliver IP therapy in an ovarian cancer setting.

Figure S1 Implant quantification.

Figure S2: Quantification of Genhance delivery through implant *in vivo*.

Table S2: Implant related complications across all *in vivo* studies.

Table S3: Preclinical study to evaluate D-luc+OVCAR-8 IP tumour growth in NSG mice, with and without the implant.

Figure S3: Tumour Growth Rate.

Table S4: Preclinical study to evaluate eNK cells + IL-15 delivered by IP injection in D-luc+OVCAR-8 NSG mouse model.

Table S5: Preclinical study to evaluate eNK cell therapies with IL-15 by IP injection in D-luc+OVCAR-8 NSG mouse model.

Table S6: Preclinical study to evaluate eNK cells + IL-15 delivered via the implant in D-luc+OVCAR-8 NSG mouse model.

Table S7: Preclinical study to evaluate eNK + IL-15 delivered via the implant in D-luc+OVCAR-8 NSG mouse model.

Figure S4: Survival and eNK efficiency outcomes of delivery modalities.

Figure S5: Implant controls tumour burden using low dose eNK + IL-15.

Table S8: Preclinical study timeline to evaluate cell sampling through the implant and low-dose eNK cell therapies with IL-15 through the implant or needle in a D-luc+OVCAR-8 NSG mouse model.

Note S1: Manufacture of replenishable peritoneal implant

Note S2: NK cell isolation and expansion

Note S3: *In vivo* studies – surgical implantation

Figure S6: Surgical Procedure

Table S2: Currently repurposed catheters are being used to deliver IP therapy in an ovarian cancer setting. Analysis of the most commonly used repurposed catheters (Tenckhoff IP catheter, Port-a-Cath, and Celsite Implantofix Access Port System) to deliver IP therapy in ovarian cancer, covering on-label use, design, repurposed use and issues in ovarian cancer.

Catheter Name	Company	Use as per Label	Design	Repurposed Use	Issues
Tenckhoff IP catheter	Medtronic	Peritoneal dialysis	Multiple side holes to facilitate even distribution of the therapeutic agent	Repurposed for IP chemotherapy due to its reliable access to the peritoneal cavity	Infection, blockage, leaks, port access problems(17, 66)
Port-a-Cath	Bard	To deliver fluids or for medical IV therapy into veins	Injection port and a flexible polyurethane catheter	Offers a more convenient and less obtrusive option for repeated IP therapy administration	Infections, blockages, leaks(18, 20)
Celsite Implantofix Access Port System	B Braun	To carry out venous infusions in a medium to long-term setting	Injection port under skin and a single hole catheter	Offers a more convenient and less obtrusive option for repeated IP therapy administration	Infections, blockages, leaks(18, 19)

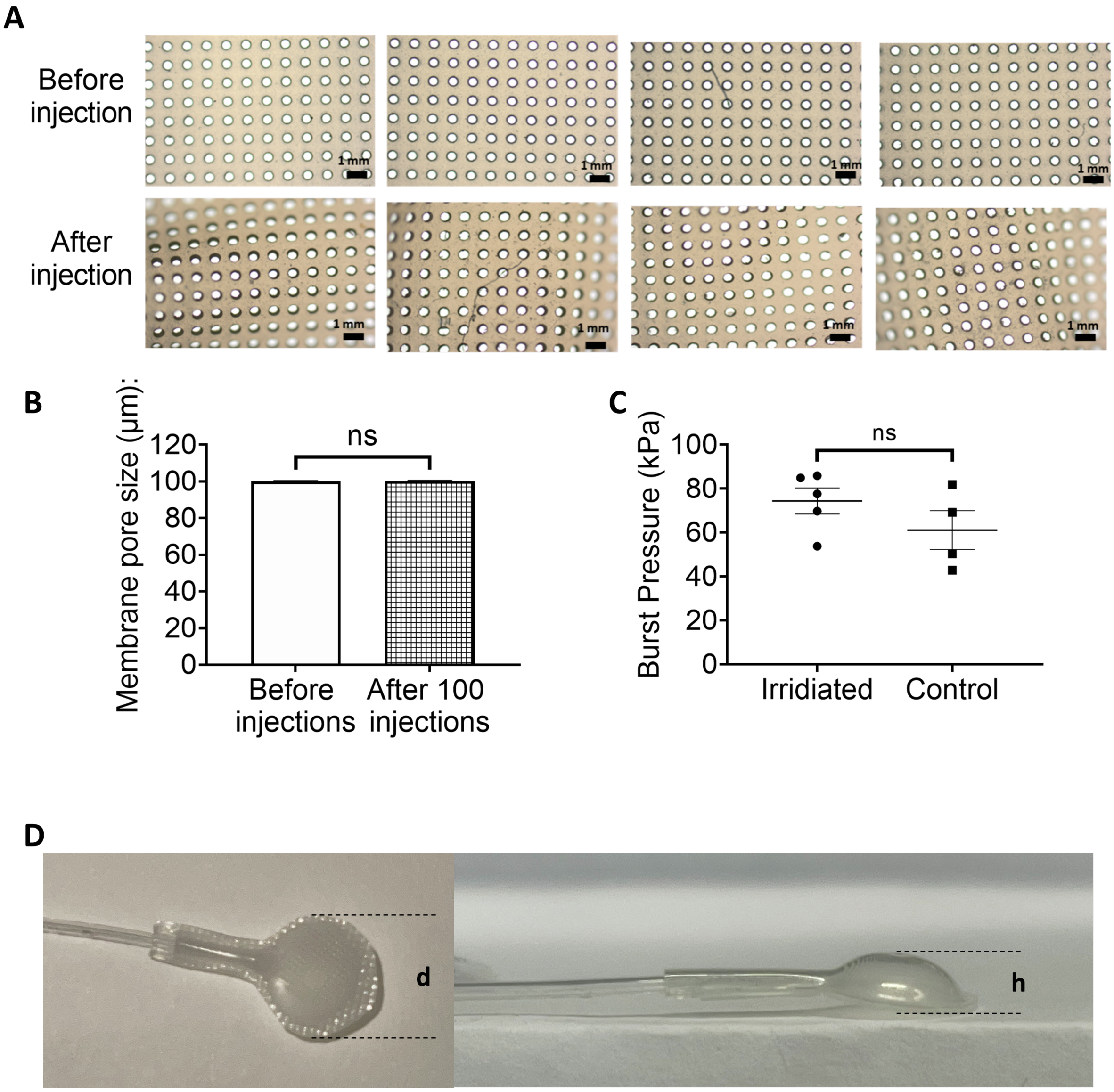

Figure S1 Implant quantification. A Light microscopy of porous membrane after 0 or 100 69 µL injections of cell culture media. Scale bar is 1 mm. **B** Pore diameters were measured after 0 or 100 injections of cell culture media. **C** Irradiated and non-irradiated non-porous implants were subject to burst pressure testing with a 1 mL/min injection speed, with no significant difference in burst pressure observed between groups. **D** The dimensions of the mouse-scaled implant were 6.5 mm diameter (**d**) and 2.5 mm height (**h**).

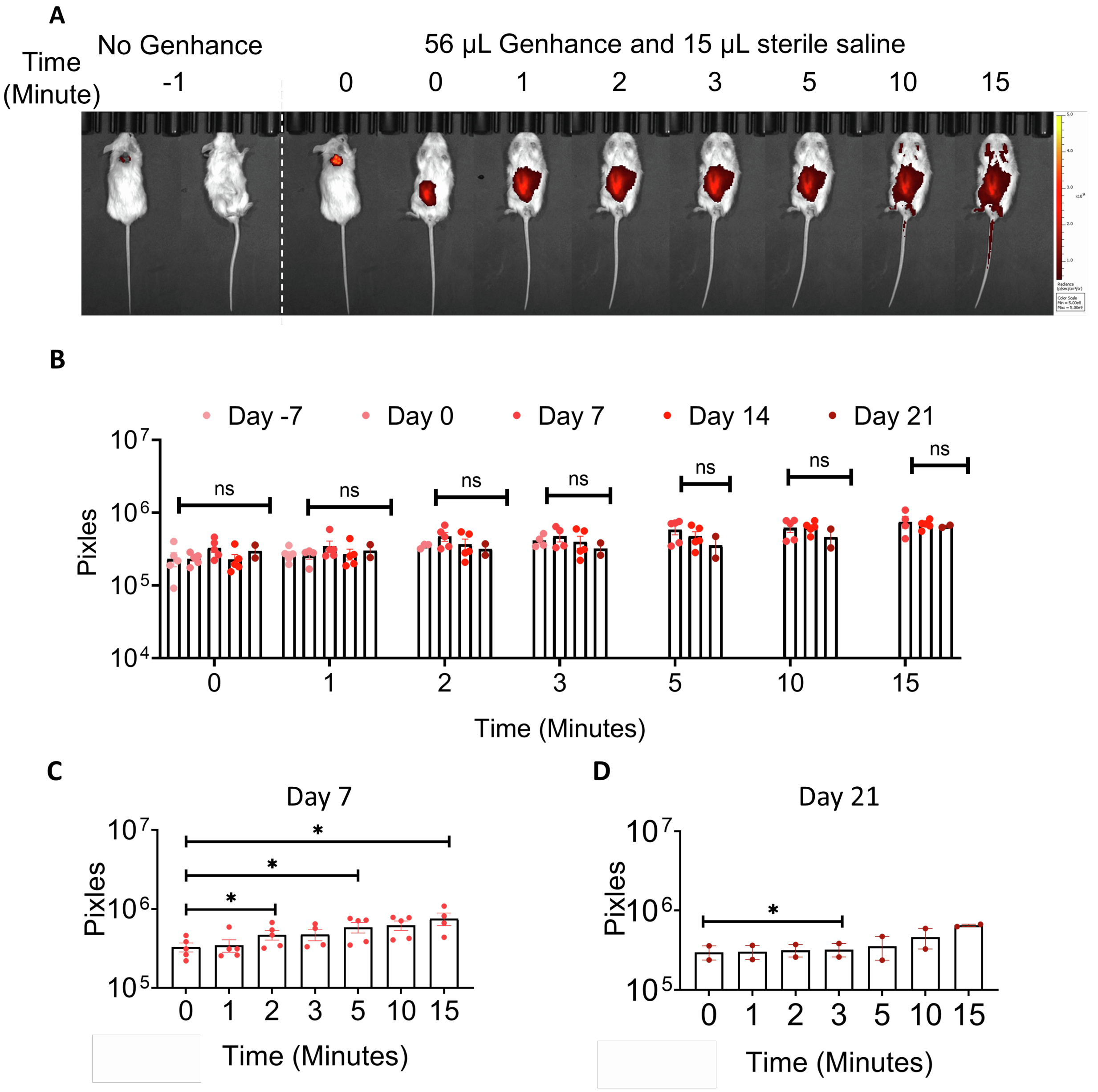

Figure S2: Quantification of Genhance delivery through implant *in vivo*. A Representative images of one mouse prior to receiving Genhance (-1 minute), after Genhance has been delivered but the line not flushed with sterile saline (0 minutes) and after the line has been flushed with sterile saline and the therapy analogue (Genhance) diffuses through the IP space (1 – 15 minutes). **B** Area of diffusion of Genhance from 0 – 15 minutes was recorded from day -7 to 21. The area of diffusion of Genhance following delivery via the implant was quantified over 15 minutes on day 7 (**C**) and day 21 (**D**).

Table S2: Implant related complications across all *in vivo* studies.

Figure	Number of mice with implant	Catheter related issues					
		Infections	Perforations	Dislodgements	Blockages	Port access problems	Other
Fig. 2	4	0	0	0	0	0	0
Fig. 3	11	0	0	0	0	0	0
Fig. 4	10	0	0	0	0	0	0
Fig. 5	8	0	0	0	0	0	0
Total	33	0	0	0	0	0	0

Table S3: Preclinical study to evaluate D-luc+OVCAR-8 IP tumour growth in NSG mice, with and without the implant. Metric: total flux radiance (p/s) on day 0 to day 35

Day	Implant Group (p/s)	Needle Group (p/s)
0	6.21e08±3.62e08	1.85e08±9.26e07
7	3.07e09±1.77e09	1.64e09±6.40e08
14	2.63e10±1.08e10	1.06e10±4.32e09
21	5.58e10±3.94e10	2.87e10±7.48e09
28	1.30e11±7.12e10	3.99e10±1.94e10
35	2.08e11±6.14e10	9.55e10±2.62e10

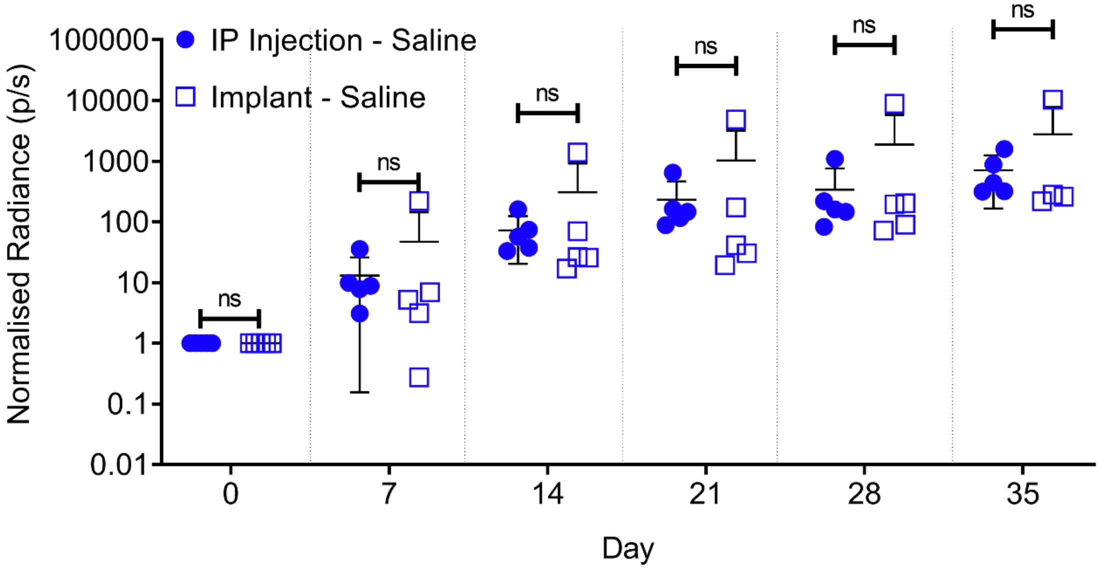

Figure S3: **Tumour Growth Rate**. To directly compare the delivery of saline (control) via the implant with IP injection at each time point, each reading was normalised to its day 0 BLI value. Data are shown as mean ± standard deviation, statistical analysis two-way ANOVA.

Table S4: Preclinical study to evaluate eNK cells + IL-15 delivered by IP injection in D-luc+OVCAR-8 NSG mouse model. Metric: total flux radiance (p/s) for each mouse on day 0-42. Control = saline delivery. 1-6 indicates animal numbers.

	D0	D7	D14	D21	D28	D35	D42
**Control1**	3.08E+08	4.93E+09	1.59E+10	1.92E+10	7.23E+08	2.60E+11	9.79E+10
**Control2**	3.63E+08	8.54E+09	5.10E+10	1.77E+10	5.58E+10	1.41E+11	X
**Control3**	2.94E+08	2.12E+09	7.12E+09	5.03E+09	3.49E+10	3.01E+10	1.12E+11
**Control4**	2.06E+08	3.37E+09	1.21E+10	1.70E+10	2.15E+10	2.69E+10	4.71E+10
**Control5**	3.26E+08	5.75E+09	9.24E+09	2.01E+10	1.91E+10	6.50E+10	8.54E+10
**Control6**	2.35E+08	4.12E+09	1.23E+10	6.85E+09	3.46E+10	1.22E+11	1.57E+11
**eNK+IL-15 1**	4.20E+08	1.05E+09	3.60E+09	5.85E+08	8.03E+08	2.05E+09	1.06E+09
**eNK+IL-15 2**	3.57E+08	1.01E+08	2.49E+08	8.41E+09	1.42E+10	3.47E+08	4.70E+08
**eNK+IL-15 3**	2.31E+08	3.06E+08	8.32E+08	1.01E+09	1.32E+09	6.14E+09	5.46E+09
**eNK+IL-15 4**	3.16E+08	1.62E+08	4.66E+08	1.19E+07	2.66E+08	1.07E+09	7.64E+08
**eNK+IL-15 5**	2.57E+08	8.31E+07	8.70E+07	1.13E+09	6.23E+08	1.28E+09	5.00E+09
**eNK+IL-15 6**	1.54E+08	7.56E+07	8.20E+07	4.10E+08	6.05E+08	2.54E+09	1.55E+10

Table S5: Preclinical study to evaluate eNK cell therapies with IL-15 by IP injection in D-luc+OVCAR-8 NSG mouse model. Metric: total flux radiance (p/s) averages on day 0-42

Day	Saline Control (p/s)	eNK + IL-15 (p/s)
0	2.89e08±5.33e07	2.89e08±8.67e07
7	4.81e09±2.02e09	2.96e08±3.46e08
14	1.79e10±1.50e10	8.86e08±1.24e08
21	1.43e10±9.03e10	1.93 e09±2.92e09
28	2.78e10±1.70e10	2.97e09±5.03e09
35	1.08e11±8.05e10	2.24e09±1.88e09
42	9.99e10±3.58e10	4.71e09±5.23e09

Table S6: Preclinical study to evaluate eNK cells + IL-15 delivered via the implant in D-luc+OVCAR-8 NSG mouse model. Metric: total flux radiance (p/s) for each mouse on day 0-42. Control = saline delivery. 1-5 indicates animal numbers. Rows highlighted in grey indicate animals that did not meet the threshold of tumour engraftment at day 0.

	D0	D7	D14	D21	D28	D35	D42
**Control1**	1.52E+09	1.73E+10	6.07E+10	2.60E+10	3.34E+11	X	X
**Control2**	1.07E+09	1.09E+10	3.83E+10	1.38E+11	3.51E+11	X	X
**Control3**	9.91E+06	3.19E+08	4.16E+08	1.12E+09	3.25E+09	X	X
**Control4**	6.90E+08	1.37E+10	2.54E+10	1.39E+11	2.98E+11	X	X
**Control5**	1.15E+09	1.02E+10	9.00E+10	3.54E+11	X	X	X
**eNK+IL-15 1**	1.69E+09	6.64E+09	4.73E+09	1.71E+09	3.22E+09	4.93E+09	3.74E+09
**eNK+IL-15 2**	1.27E+09	7.07E+08	3.05E+08	2.04E+08	4.77E+08	7.50E+08	1.35E+09
**eNK+IL-15 3**	3.75E+06	1.00E+07	5.05E+06	3.63E+06	1.45E+07	2.44E+07	1.01E+07
**eNK+IL-15 4**	1.04E+09	1.52E+07	4.13E+07	3.03E+08	1.75E+09	1.81E+09	1.49E+09
**eNK+IL-15 5**	9.04E+08	9.02E+08	1.49E+09	2.71E+07	1.18E+10	4.25E+07	4.26E+08

Table S7: Preclinical study to evaluate eNK + IL-15 delivered via the implant in D-luc+OVCAR-8 NSG mouse model. Metric: total flux radiance (p/s) averages on day 0-42

**Day**	**Saline Control (p/s)**	**eNK + IL-15 (p/s)**
0	1.08e09±3.40e08	1.23e09±2.09e08
7	2.07e09±2.66e09	1.07e10±4.54e09
14	5.36e10±2.45e10	1.58e09±1.91e09
21	1.64e11±1.19e11	1.23e09±1.04e09
28	3.28e11±2.21e10	1.69e09±9.95e08
35	X	1.88e09±1.87e09
42	X	1.75e09±1.22+09

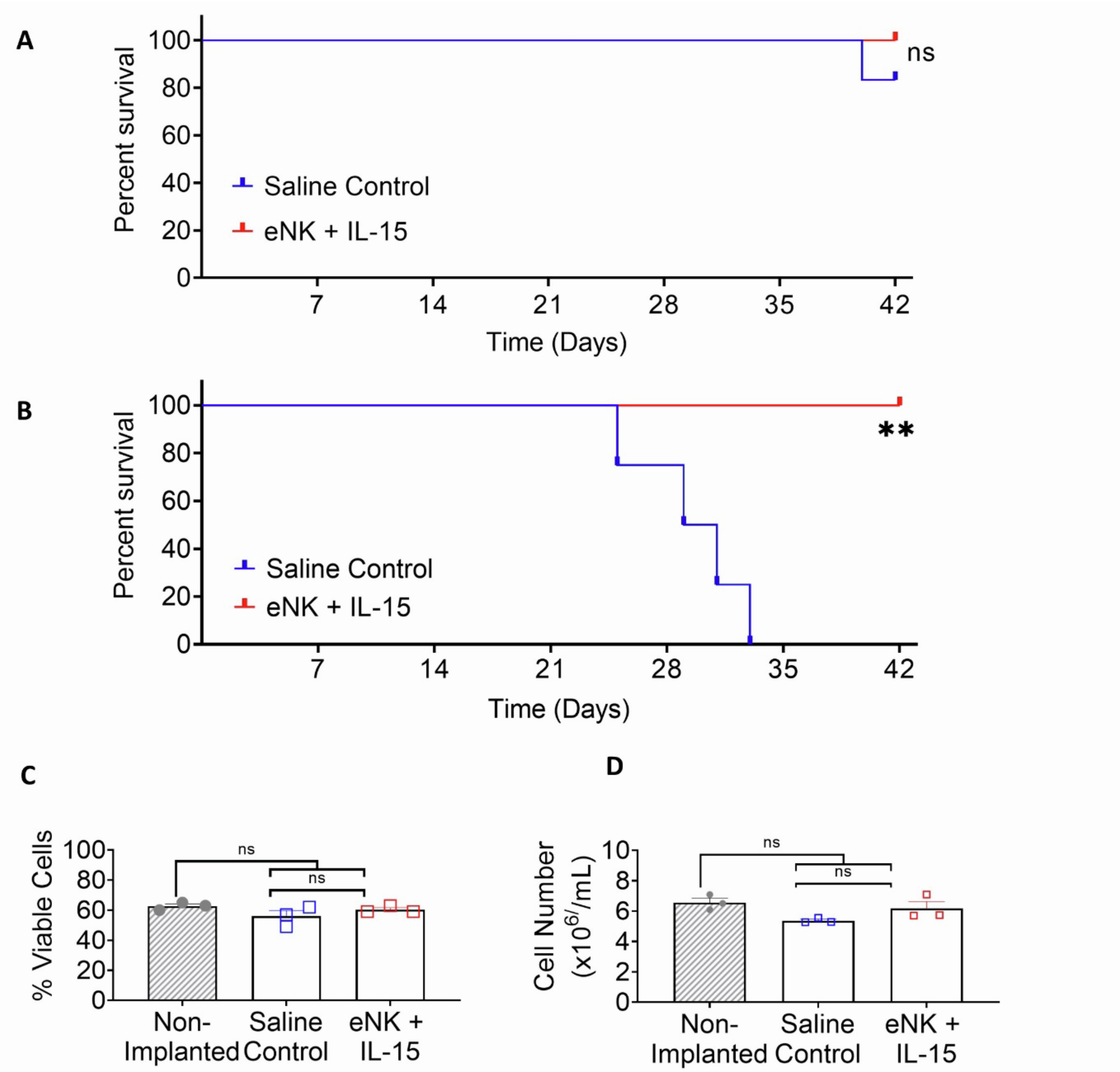

Figure S4: Survival and eNK efficiency outcomes of delivery modalities. **A** Survival curve comparing IP injection of saline (control) versus IP injection of eNK cells + IL-15 (therapeutic regime). No significant survival benefit was observed in the therapeutic group compared with saline controls, as determined through log-rank (Mantel-Cox) test **B** Survival curve comparing saline (control) delivered via implant versus implant delivery of eNK + IL-15 (therapeutic regime). A significant survival benefit was observed in the therapeutic group ($p=0.0067$) as determined through log-rank (Mantel-Cox) test. Post-euthanasia at 42 days, implants were explanted and tested for functionality. eNK cells were delivered to new, non-implanted implants vs explanted implants from the saline control group and explanted implants from the therapy group. Cell viability (**C**) and cell number (**D**) were quantified.

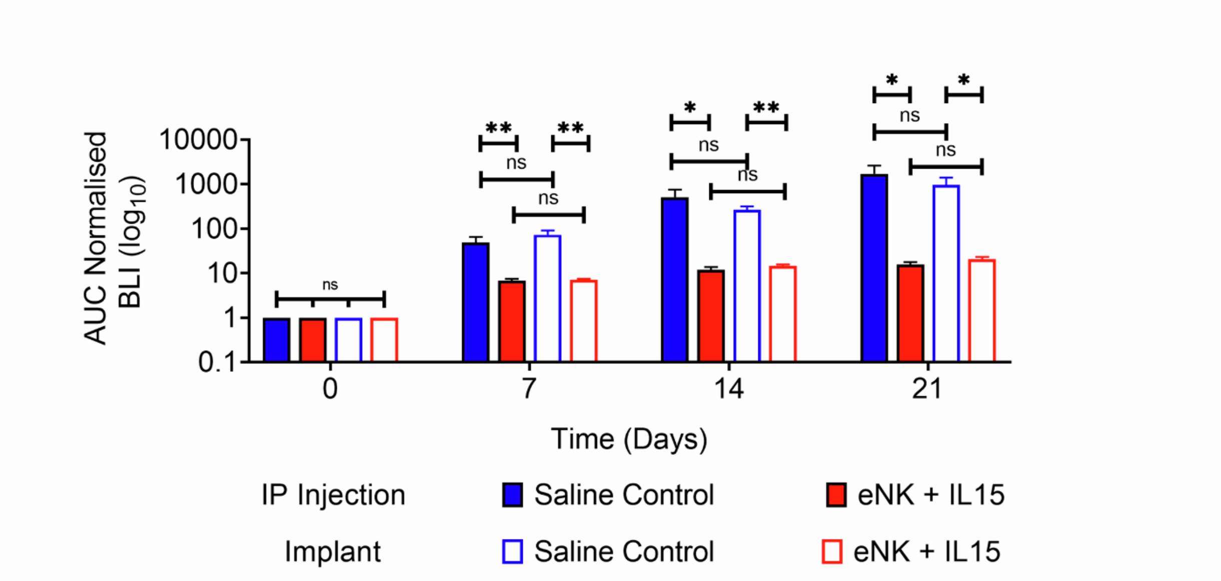

Figure S5: Implant controls tumour burden using low dose eNK + IL-15. To directly compare the delivery of saline (control) or eNK cells + IL-15 (treatment) via our implant or through IP injection at each time point, we normalised each reading to its day 0 BLI value and the area under the curve (AUC) was quantified, denoting overall functional effect over the study duration.

Table S8*: Preclinical study timeline to evaluate cell sampling through the implant and low-dose eNK cell therapies with IL-15 through the implant or needle in a D-luc+OVCAR-8 NSG mouse model.* Metric: area under normalised radiance (p/s) curve AUC denoting overall functional effect mapped over study duration.

	**No Implant**	**Implant**
**Day**	*eNK + IL-15 AUC*	*eNK + IL-15 AUC*
0-7	6.945±2.964	8.328±5.288
0-14	20.01±16.921	17.115±14.394
0-21	55.096±30.343	24.979±19.908
0-28	118.196±100.5	33.62±23.325
0-35	193.542±138.177	42.985±24.468
42	334.58±155.555	52.155±29.977

Note S1: Manufacture of replenishable peritoneal implant

The dimensions of the mouse-scaled implant were 2.5 mm height and 6.5 mm diameter, connected to a catheter with an inner diameter = 0.305 mm, outer diameter = 0.635 mm and length = 5 cm) with a transcutaneous port suitable for repeated access. The volume of the reservoir was 56 µL and the dead volume in the system was 15 µL (total volume = 71 µL).

Therapeutic reservoirs were manufactured from TPU polyether film (American Polyfilm, Inc.). Porous membranes were manufactured using a TroTec Speedy 100 laser. CorelDraw software (Standard 2021, 64-bit) was used to generate the pattern to be laser cut. A 5 mm porous region with 100 μm diameter pores at a total porosity of 10 % was achieved using a 0.29 mm centre-to-centre pore spacing. TPU sheets (0.15 mm thickness) were placed on the laser bed, and the previously generated pore design was cut using the following parameters: power = 18%, speed = 0.4%, frequency = 2000 Hz. Scanning electron microscopy (SEM) was used to image the membranes for quality control (magnification = 180x, accelerating voltage = 15.0 kV), and their porosity and mean pore size were quantified using Fiji (Image J). Next, the reservoirs were assembled following previously established techniques[42]. A hemispherical reservoir (diameter = 6.5 mm, height = 2 mm) was formed through thermal forming 0.3048 mm thick TPU sheets over custom 3D printed moulds using a Yescom Dental Vacuum Former, JT-18, 220 V, 800 W. Micro-renethane catheter tubing (MRE025, Braintree Scientific, with diameter = 0.635 mm and length = 5 cm) was inserted between the hemispherical reservoirs and the porous membranes, and the three components were heat sealed together using a heat transfer machine (QX-A1, 110V, 1800W, PowerPress) to form a single assembly. Each assembly was sterilized using Ethylene Oxide. Prior to implantation, catheters were connected to a self-sealing transcutaneous access port (VABM1B/22, Instech Laboratories).

Note S2: NK cell isolation and expansion

Deidentified human blood products were obtained from Memorial Blood Bank (Minneapolis, MN). Their use was approved by the University of Minnesota and National Marrow Donor Program institutional review board (ID9709M00134) in accordance with the Declaration of Helsinki. Donor information available normal male donor blood from Memorial Blood Centers.

Blood was processed to obtain PBMCs using density gradient Ficoll-Paque (GE Healthcare, catalog no. GE17-5442-03). For experiments with enriched NK cells (eNK), PBMCs were processed fresh using the EasySep Human NK Cell Enrichment Kit (STEMCELL Technologies, catalog no. 19055). NK cells were expanded as previously described by Denman et al.,[3] whereby irradiated K562 feeder cells engineered to express 4-1BBL and membrane-bound IL-21 were utilized as feeder cell. NK cells were expanded over a 2 week period and population confirmed using flow cytometry with markers CD56 and CD3.

Note S3: *In vivo* studies – surgical implantation

For surgical placement of the implant a pre-operative analgesic, Carprofen was administered via subcutaneous injection (SQ) 5mg/kg (dosage has since changed to 10mg/kg due to IACUC updates). The regional nerve blocker Lidocaine 4mg/kg was injected locally at the surgical sites prior to surgery. Anaesthesia was induced using isoflurane (5% induction, 1-2% maintenance). Anaesthesia was confirmed using a toe pinch. All relevant areas of the mouse, including the abdomen, left flank, and between the shoulder blades, were shaved and residual hair was removed using Nair™ epilatory cream. These areas were cleaned with iodine followed by 70% ethanol, repeated three times. In a sterile environment, mice were placed on sterile drapes in the prone position. A subcutaneous horizontal incision (1-1.5 cm) was made at the base of the neck to create a pocket for the transcutaneous port (Fig. S6, A). Mice were moved into the supine position to access the abdomen. A 1-1.5 cm incision was made between the ribcage and the top of the right hind leg for implant insertion (Fig. S6, B). Blunt dissection was used to tunnel from the neck to the abdominal incision, leaving space for the transcutaneous port and catheter tubing (Fig. S6, C, D). The transcutaneous port was then tunnelled from the abdominal incision to the neck incision (Fig. S6, E, F). Next, a 1 cm incision in the peritoneal wall was made through the abdominal incision (Fig. S6, G). The porous reservoir, connected to the catheter, was positioned away from the fatty tissue and below the liver, with the porous membranes facing the internal organs (Fig. S6, H, I). The catheter was brought to the one side of the peritoneal incision, anchored using two sutures and the incision was closed with internal suture(s) to secure the reservoir (Fig. S6, J). The abdominal skin incision was then closed with two or three surgical staples, while two or three interrupted sutures were used to secure the transcutaneous port in place and close the subcutaneous incision (Fig. S6, K). The port was secured and neck incision was then closed with two or three interrupted sutures on either side of the port (Fig. S6, L, M). The implant was flushed with 500 µL of sterile saline and 1 mL of sterile saline was administered subcutaneously in multiple boluses for rehydration. Post-surgery, mice are given ophthalmic ointment. Mice are monitored post-operatively for three consecutive days after surgery with daily carprofen SQ.

Pocket formation

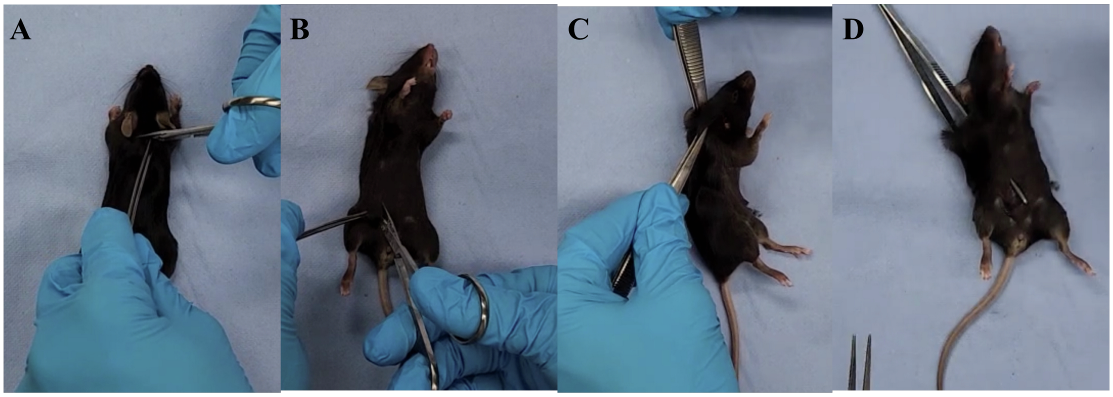

Port placement at nape of the neck

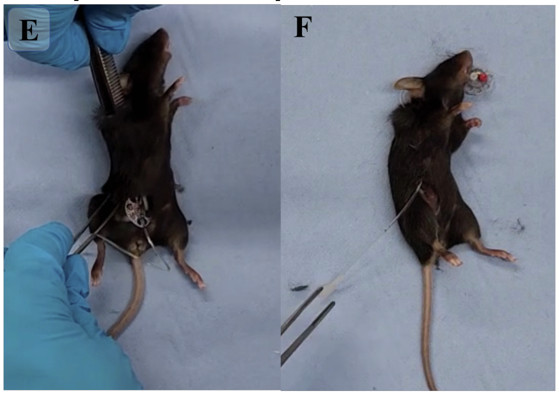

Reservoir placement within the intraperitoneal cavity

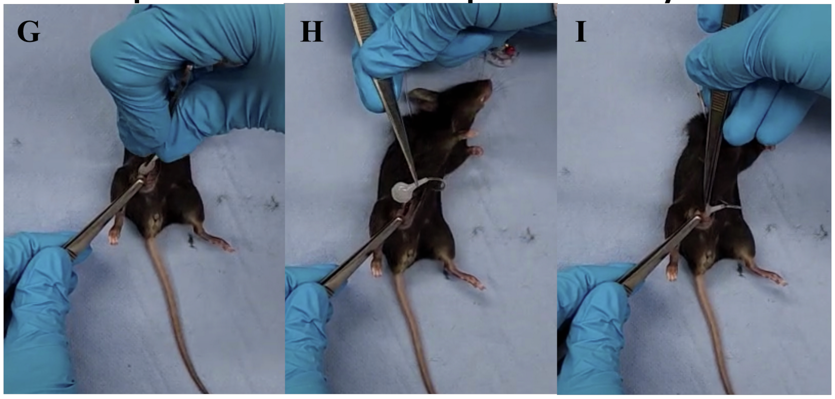

Closing of cavities internally and externally, and securing transcutaneous access port

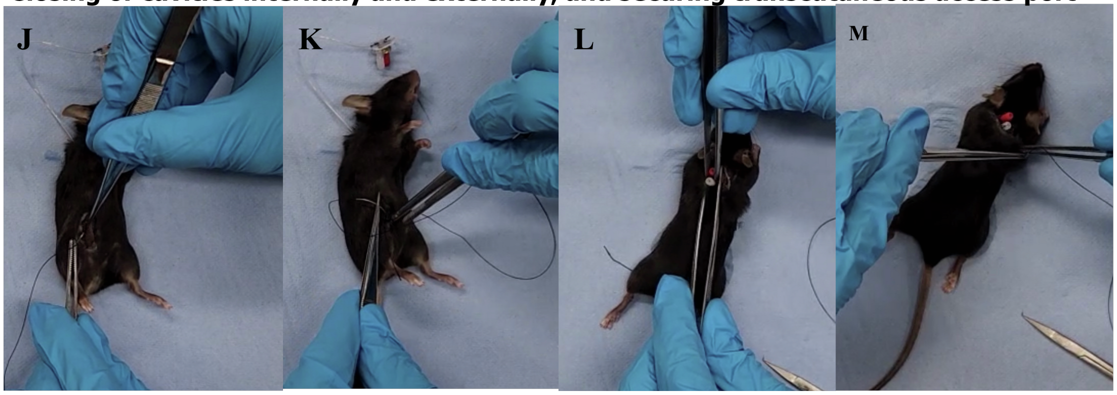

Figure S6: Surgical Procedure: A subcutaneous horizontal incision (1-1.5 cm) was made at the base of the neck to create a pocket for the transcutaneous port (A). Mice were moved into the supine position to access the abdomen. A 1-1.5 cm incision was made between the ribcage and the top of the right hind leg for implant insertion (B). Blunt dissection was used to tunnel from the neck to the abdominal incision, leaving space for the transcutaneous port and catheter tubing (C, D). The transcutaneous port was then tunneled from the abdominal incision to the neck incision (E, F). Next, a 1 cm incision in the peritoneal wall was made through the abdominal incision (G). The porous reservoir, connected to the catheter, was positioned away from the fatty tissue and below the liver, with the porous membranes facing the internal organs (H, I). The catheter was brought to the one side of the peritoneal incision, anchored using two sutures and the incision was closed with internal suture(s) to secure the reservoir (J). The abdominal skin incision was then closed with two or three surgical staples, while two or three interrupted sutures were used to secure the transcutaneous port in place and close the subcutaneous incision (K). The port was secured and neck incision was then closed with two or three interrupted sutures on either side of the port (L, M). C57black6 mouse was used for the purpose of generating this figure.
